# Recent Genetic Gains in Nitrogen Use Efficiency in Oilseed Rape

**DOI:** 10.3389/fpls.2017.00963

**Published:** 2017-06-07

**Authors:** Andreas Stahl, Mara Pfeifer, Matthias Frisch, Benjamin Wittkop, Rod J. Snowdon

**Affiliations:** ^1^Department of Plant Breeding, IFZ Research Centre for Biosystems, Land Use and Nutrition, Justus Liebig University GiessenGiessen, Germany; ^2^Department of Biometry and Population Genetics, IFZ Research Centre for Biosystems, Land Use and Nutrition, Justus Liebig University GiessenGiessen, Germany

**Keywords:** yield, breeding progress, *Brassica napus*, hybrid varieties, nitrogen, fertilization, oil, sustainable intensification

## Abstract

Nitrogen is essential for plant growth, and N fertilization allows farmers to obtain high yields and produce sufficient agricultural commodities. On the other hand, nitrogen losses potentially cause adverse effects to ecosystems and to human health. Increasing nitrogen use efficiency (NUE) is vital to solve the conflict between productivity, to secure the demand of a growing world population, and the protection of the environment. To ensure this, genetic improvement is considered to be a paramount aspect toward ecofriendly crop production. Winter oilseed rape (*Brassica napus* L.) is the second most important oilseed crop in the world and is cultivated in many regions across the temperate zones. To our knowledge, this study reports the most comprehensive field-based data generated to date for an empirical evaluation of genetic improvement in winter oilseed rape varieties under two divergent nitrogen fertilization levels (NFLs). A collection of 30 elite varieties registered between 1989 and 2014, including hybrids and open pollinated varieties, was tested in a 2-year experiment in 10 environments across Germany for changes in seed yield and seed quality traits. Furthermore, NUE was calculated. We observed a highly significant genetics-driven increase in seed yield *per*-*se* and, thus, increased NUE at both NFLs. On average, seed yield from modern open-pollinated varieties and modern hybrids was higher than from old open-pollinated varieties and old hybrids. The annual yield progress across all tested varieties was ~35 kg ha^−1^ year^−1^ at low nitrogen and 45 kg ha^−1^ year^−1^ under high nitrogen fertilization. Furthermore, in modern varieties an increased oil concentration and decreased protein concentration was observed. Despite, the significant effects of nitrogen fertilization, a surprisingly low average seed yield gap of 180 kg N ha^−1^ was noted between high and low nitrogen fertilization. Due to contrary effects of N fertilization on seed yield *per*-*se* and seed oil concentration an oil yield of 2.04 t ha^−1^ was measured at both N levels. Collectively, the data reveal that genetic improvement through modern breeding techniques in conjunction with reduced N fertilizer inputs has a tremendous potential to increase NUE of oilseed rape.

## Introduction

An increase in global crop demand of up to 110% is expected by 2050 compared to 2005, requiring a tremendous increase in production (Tilman et al., 2011). Competitive crop production crucially depends on the adequate application of nitrogen (N). It is well-known that the application of N fertilizer is a substantial driver of yield increases in the last century. At the same time, it has also been found that 50–70% of the applied N is not recovered in the harvested plant organs (seeds) and can cause severe damages to the surrounding ecosystems (Sylvester-Bradley and Kindred, 2009; Liu et al., 2010; Galloway et al., 2013). Estimations have revealed that the current global conversion of atmospheric N_2_ into reactive N and its application on fields have already transgressed the boundaries of sustainable development (Rockström et al., 2009; Steffen et al., 2015). Moreover, the lost N is not only an ecological problem but also uneconomical for farmers (Rothstein, 2007). Therefore, in the decades ahead, agricultural crop production faces the unprecedented challenge of enhancing crop yields to match the increasing demands, while simultaneously reducing environmental damages caused by unused nitrogen. To master this dichotomy, a dramatic increase in nitrogen use efficiency (NUE) is inevitable. Besides a more precise fertilizer application (Henke et al., 2008; Müller et al., 2012), the use of genetic potential by breeding and cultivation of most suitable and efficient varieties is considered to play an important role in the sustainable intensification of agriculture (Hirel et al., 2007; Kant et al., 2011; Hawkesford, 2014).

Oilseed rape (*Brassica napus* L.) is the principle European oil crop and the second most important oilseed crop in the world after soybean, with a wide dissemination across countries in the moderate climatic zone (Fischer et al., 2014). It is mainly cultivated because of its high quality vegetable oil used for human nutrition purposes (Kumar et al., 2016) and as a renewable source for fuels and technical oils. Not at least, the residual meal is winning wide use as a protein feed for animals and is also discussed for human nutrition recently (Fleddermann et al., 2013). Despite several positive agronomic effects, including its integral role as a break crop in cereal crop rotations, its ability to improve soil fertility, and its strong ability to capture nutrients during vegetative growth stages, oilseed rape cultivation is often associated with a relatively high N balance surplus (Sieling and Kage, 2006; Weiser et al., 2017). Consequently, a more competitive and eco-friendly production of oil and protein would benefit from increased NUE.

In the face of this challenge the question arises whether or not breeding is directing NUE improvement. A critical assessment of the genetic progress for SY under divergent N inputs is of great value in order to analyze the extent of the breeding effect on NUE improvement in the past and in what way a course correction in breeding programs is required to reach future sustainability goals. Breeding is a long-term procedure and environmental conditions as well as management practices have also changed over time. Therefore, the isolation of the genetic contribution by comparing the direct performance between varieties from different period of registration requires multilocation field trials with simultaneous cultivation of the varieties under a very similar N fertilization management. Owing to the tedious trial setup, experimental data of those comparisons are seldom presented.

In the case of oilseed rape Kessel et al. (2012) evaluated 36 genotypes in the vegetation period 1997–1998. This study included 3 hybrids, 6 resynthesized lines, 8 old cultivars, and 19 modern cultivars. It was found that modern cultivars and hybrids outperform older varieties and resynthesized lines. However, the varieties designated as modern in that study no longer have any relevance for present farming practice. Moreover, since hybrids varieties have gained enormous importance in the last two decades, today comprising the vast majority of available varieties, this type of variety must be considered for yield and NUE monitoring with an up-to-date European benchmark. In this regard, Rathke and Diepenbrock (2006) investigated the energy balance of winter oilseed rape depending on the N fertilizer inputs and suggested that SY levels should be assessed through further studies to document progress in plant breeding including the advent of hybrid varieties. Although further field studies on NUE in oilseed rape have since been conducted, most focussed on genetic mapping approaches, either in experimental populations (Bouchet et al., 2014; Nyikako et al., 2014; Miersch et al., 2016) or in case of association studies (Bouchet et al., 2016) do not address the breeding progress.

The present study aims to (i) assess the SY change through breeding during the last decades, (ii) examine the response to nitrogen of older and modern varieties in multilocation field trials, (iii) investigate the change and interrelationship of seed quality traits affected by environment, N fertilization, and breeding activities, and (iv) provide evidence-based suggestions for further orientations in oilseed rape breeding and cultivation.

## Materials and methods

### Plant material

Diverse varieties (*n* = 30) from different breeding companies and a period of registration between 1989 and 2014 were investigated (Table 1). All of them were selected varieties that were adapted to the northern European growing conditions and have passed the official varieties test by official varieties agency. In order to keep the number of genotype on a manageable size, major varieties from the different periods of registration were selected as proposed in a similar study on wheat by Lopes et al. (2012). According to the year of registration, varieties were grouped as older and modern varieties.

**Table 1:** Groups of investigated varieties according to year of registration and type of variety.

**Varieties**	**Year of registration**
**NEW HYBRID VARIETIES**	
Thure (SDH)	2014
Marathon	2013
Mercedes	2013
Avatar	2011
DK Exstorm	2011
Inspiration	2011
Genie	2011
Mascara	2011
Troy (SDH)	2011
Artoga	2010
Sherpa	2010
Compass	2009
NK Linus	2009
Visby	2007
**OLD HYBRID VARIETIES**	
Exocet	2005
Taurus	2004
Baldur	2002
Elektra	2002
Ryder	2000
Artus	1997
**NEW OP VARIETIES**	
Patron	2012
Trinity	2012
Adriana	2007
Lorenz	2005
Oase	2004
**OLD OP VARIETIES**	
Pacific	2003
Californium	2002
Aviso	2000
Express	1993
Lirajet	1989

*SDH, Semi dwarf hybrid*.

### Field experiments

Since environmental factors heavily affect the phenotype, 10 experiments were conducted in total at six different locations in Germany during two subsequent growing seasons (Table 2). Environmental specifications are given in Supplementary Tables 1, 2. Experiments were conducted in a split plot design for N treatments. Within each nitrogen fertilization level (NFL, as main plot), the genotypes were arranged in three replicates with eight sub blocks each, according to an alpha lattice designed with R package *agricolae* (De Mendiburu and Simon, 2015). The sowing rate was adjusted for the previous tested germination rate to target a final plant density of 50 plants per square meter. At all locations, a plot in plot system was used, and only the middle part of each plot was harvested in order to avoid side effects from the neighboring plots. Organic fertilizers were applied neither during the experiment nor during cultivation of the preceding crops. Moreover, no intercrop was cultivated before the experiment. In the first year of experiment, 120 kg N ha^−1^ was applied in both low N (LN) and high N treatment (HN) at the beginning of the spring vegetation. Another 100 kg N ha^−1^ was applied only in HN during bolting. In the second year, 65 kg N ha^−1^ was applied in LN and 120 kg N ha^−1^ in HN at the first application. The second application during bolting comprised of 55 kg N ha^−1^ in LN and 100 kg N ha^−1^ in HN. In both years, the first application of fertilizer was reduced for soil mineral N (N_min_) present in the soil at the end of winter (Supplementary Table 1). The intention of the high N application was to simulate intensive crop production conditions as they are common in Northern Europe. The low N treatment was to investigate the response of the genotypes during reduced fertilizer application. All other nutrients were applied at the same level across both NFL at the particular locations. Furthermore, full control of weeds, pest (insects), and diseases (fungi) and straw stiffness were conducted at each side on a local appropriate level. Thus, only N was varied and all other factors were kept on an intensive level in both NFL levels.

**Table 2:** Overview of single experiments and side specific conditions.

**Experiment**	**Location**	**GPS Position**	**Date of sowing**	**Date of harvest**	**Preceding crop/Pre-preceding crop**
ASE15	Asendorf	52.763480, 8.996599	26.08.2014	01.08.2015	Winterbarley/Winterwheat
ASE16	Asendorf	52.719760, 8.962428	26.08.2015	20/21.07.2016	Winterbarley/Winterwheat
BOV16	Bovenau	54.360205, 9.806736	26.08.2015	24.07.2016	Winter Barley/Winter Barley
MOS15	Moosburg	48.500523, 11.936888	25.08.2014	16.07.2015	Winterwheat/Potatos
MOS16	Moosburg	48.500473, 11.941212	28.08.2015	20.07.2016	Winterbarley/Winterwheat
NIE16	Nienstädt	52.260731, 9.091446	04.09.2015	14.07.2016	Winterbarley/Winterwheat
RHH15	Rauischholzhausen	50.779556, 8.889699	03.09.2014	21/22.07.2015	Winterwheat/Maize
RHH16	Rauischholzhausen	50.778334, 8.868113	26.08.2015	21.07.2016	Winterwheat/Maize
ROS15	Rosenthal	52.306046, 10.164338	25.08.2014	21.07.2015	Winterwheat/Winterwheat
ROS16	Rosenthal	52.298665, 10.117582	25.08.2015	19.07.2016	Winterwheat/Winterwheat

### Data collection

Seed yield was determined by threshing from standing mature canopy in the second half of July or first days of August (Table 2). The water content of seeds was determined immediately after seed harvest, and SY was corrected to a standard water content of 9%. From each individual plot, an aliquot of at least 100 g were used to determine seed oil concentration (OilConc) and seed protein concentration (ProteinConc) in duplicates on the same machine (Unity SpectraStar 2500, Brookfield, USA) via near-infrared reflectance spectroscopy (Tkachuk, 1981; Reinhardt, 1992; Tillmann and Paul, 1998; Tillmann et al., 2000). Subsequently, OilConc and ProteinConc were also corrected to 9% water content and multiplied with SY in order to determine seed oil yield (OilY) and seed protein yield (ProteinY). Since one old hybrid variety showed a strongly reduced germination rate at all locations in the second experimental year, it was excluded from further data analysis.

In environments ASE15, ASE16, NIE16, and MOS16, lodging was evaluated before harvest on each individual plot on a scale from 1 (no lodging at all) to 9 (plot is completely lodging).

### Data analysis

Adjusted means for each variety at each NFL across all locations were estimated using a linear model with varieties, NFL, and their interaction as fixed factors; and interactions of year, location, NFL, replicate, and block considered as random factors (Equation 1). *P*-values for significance of fixed effects were derived from an analysis of variance (ANOVA) using the models described in Equation (1).

(1) $$\begin{array}{l} {\text{P}}_{ijklmn}=\mu +g_{i}+n_{j}+gn_{ij}+{\text{B}}_{jklmn}+{\text{R}}_{jkmn}+{\text{W}}_{jkm}\\ \;+\;{\text{E}}_{km}+{\text{e}}_{ijklmn} \end{array}$$

with P_*ijklmn*_ as the observed phenotype of the *i*th variety, the *j*th nitrogen fertilization, the *k*th year, the *m*th location, the *n*th replicate, and the *l*th block. μ is the general mean of the experiment, **g**_i_ is the *i*th fixed effect of variety, ***n***_*j*_ is the *j*th fixed effect of NFL, and ***gn***_*ij*_ is the fixed effect of variety by NFL interaction. B_*jklmn*_ is the random effect of the *l*th block within the *n*th replicate, within the *j*th main plot, at the *m*th location, and at the *k*th year. R_*jkmn*_ is the random effect of the *n*th replicate, within the *j*th main plot, at the *m*th location, and at the *k*th year. W_*jkm*_ is the random effect of the *j*th main plot, at the *m*th location, and at the *k*th year. E_*km*_ is the random effect of the environment at the *m*th location in the *k*th year. e_*ijklmn*_ is the error term. Fixed effects are written in bold lowercase letters.

To estimate the adjusted means of each variety at individual year (Equation 2) and location (Equation 3), the linear model was modified and only the interactions of the location, main plot, replicate, and block were considered to be random factors. Fixed effects are written in bold lowercase letters.

(2) $$\begin{array}{l} {\text{P}}_{ijlmn}=\mu +g_{i}+n_{j}+gn_{ij}+{\text{B}}_{jlmn}+{\text{R}}_{jmn}+{\text{W}}_{jm}\\ \;+\;{\text{E}}_{km}+{\text{e}}_{ijlmn} \end{array}$$

(3) $${\text{P}}_{ijln}=\mu +g_{i}+n_{j}+gn_{ij}+{\text{B}}_{jln}+{\text{R}}_{jn}+{\text{W}}_{j}+{\text{e}}_{ijln}$$

The model shown in Equation (4) was used to estimate the variance for broad sense heritability estimation. In contrast to Equation (1), **g**_*i*_ is the *i*th random effect of variety, ***n***_*j*_ is the *j*th fixed effect of NFL, and ***gn***_*ij*_ is the random effect of variety by NFL interaction. GE_*ikm*_ is the random effect of the G × E interaction. Fixed effect is written in bold. Equation (5) was used to estimate the broad sense heritability.

(4) $$\begin{array}{l} {\text{P}}_{ijklmn}=\mu +\;G_{i}+n_{j}+{\text{GN}}_{ij}+{\text{GE}}_{ikm}+{\text{B}}_{jklmn}+{\text{R}}_{jkmn}\\ \;+\;{\text{W}}_{jkm}+E_{km}+e_{ijklmn} \end{array}$$

(5) $$\;h^{2}=\frac{\sigma_{G}^{2}}{\sigma_{G}^{2}+\frac{\sigma_{G\;\text{ x}\;N}^{2}}{\;j\;}+\frac{\sigma_{G\;\text{x}\;\text{E}\;}^{2}}{p\;}\;+\frac{\sigma_{\text{e}}^{2}}{j\;x\;p\;x\;l\;x\;n\;}}$$

with j the number of NFL, p the number of the investigated environments, l the number of blocks, and n the number of replicates.

All analyses were conducted by statistical software R (R Core Team, 2013) by using the packages lmerTest (Kuznetsova et al., 2016), lsmeans (Lenth, 2016), and lme4 (Bates et al., 2015).

Adjusted data across all environments were used to determine the NUE, which is expressed as SY over N fertilizer application. In addition, the amount of N fertilization required to produce 1 ton of rapeseed oil was determined. Therefore, 1 ton was divided by the particular OilY of individual varieties and multiplied with the NFL. Production losses and inefficiency in post-harvest proceedings were neglected in this calculation.

Pairwise Pearson correlation coefficients (r) were estimated between individual traits and year of registration, as well as between the trait value observed at HN and LN.

Packages ggplot2 were used to design diagrams (Wickham, 2009).

## Results

### Seed yield

Across all 10 environments, SY varied between 3.79 and 5.00 t ha^−1^ for LN and between 3.91 and 5.13 t ha^−1^ for HN (Table 3). At some location the variation was even bigger (Supplementary Table 4). The average SY in experimental year 2014–2015 was about 220 kg and 480 kg ha^−1^ higher in LN and HN respectively, compared to the experimental year 2015–2016. While the genetic effect on yield was highly significant in each of the environments, except RHH15 (*p* = 0.0181), NFL was only significant in some environments (Supplementary Table 4). Over all tested environments, difference in N fertilization resulted in a yield difference of 180 kg ha^−1^, which was only significant on the 10% error level, according to ANOVA. G × N interaction was significant only at several individual environments (MOS and ASE in both years, Supplementary Table 4). Generally, the correlation between the environments at HN is high, ranking between *r* = 0.17 and *r* = 0.81, and was significant in most cases (Supplementary Figure 1B). At LN, it was observed that the environment RHH15 did not show significant correlation to most other environment, while all the other environments were positive and significantly correlated to each other. The highest correlation between environments was *r* = 0.77 (Supplementary Figure 1A).

**Table 3:** Descriptive statistics for investigated traits

		**Low nitrogen fertilization**				**High nitrogen fertilization**			
		**Min**	**Max**	**Mean**	**CoV**	**Min**	**Max**	**Mean**	**CoV**
Seed Yield (t/ha)	Mean 2015	3.94	4.94	4.60	0.05	4.00	5.39	4.94	0.07
	Mean 2016	3.70	5.06	4.38	0.07	3.67	5.06	4.46	0.07
	Mean	3.79	5.00	4.47	0.06	3.91	5.13	4.65	0.07
NUE (kg/kg)	Mean 2015	32.86	41.13	38.32	0.05	18.19	24.49	22.46	0.07
	Mean 2016	30.81	42.19	36.53	0.07	16.66	23.01	20.26	0.07
	Mean	31.56	41.70	37.25	0.06	17.76	23.34	21.12	0.07
Protein Conc (%)	Mean 2015	13.90	15.98	14.97	0.04	15.97	18.09	16.89	0.04
	Mean 2016	14.90	17.09	15.73	0.04	16.61	18.49	17.43	0.03
	Mean	14.64	16.52	15.43	0.03	16.35	18.28	17.22	0.03
Oil Conc (%)	Mean 2015	43.96	47.97	45.80	0.02	41.48	45.84	43.96	0.03
	Mean 2016	43.47	47.57	45.55	0.02	41.60	45.67	44.00	0.02
	Mean	43.72	47.56	45.64	0.02	41.53	45.64	43.96	0.02
Protein Yield (t/ha)	Mean 2015	0.62	0.74	0.69	0.05	0.69	0.93	0.84	0.06
	Mean 2016	0.62	0.78	0.69	0.06	0.67	0.89	0.78	0.06
	Mean	0.62	0.76	0.69	0.05	0.70	0.89	0.80	0.06
Oil Yield (t/ha)	Mean 2015	1.80	2.29	2.11	0.06	1.69	2.40	2.17	0.08
	Mean 2016	1.68	2.33	1.99	0.08	1.59	2.24	1.96	0.08
	Mean	1.73	2.30	2.04	0.07	1.63	2.27	2.04	0.08
Protein Conc DFF (%)	Mean 2015	26.28	29.50	27.55	0.02	28.78	32.29	30.09	0.03
	Mean 2016	28.01	30.60	28.86	0.02	30.19	32.76	31.12	0.02
	Mean	27.27	30.09	28.29	0.02	29.67	32.49	30.67	0.02

*Adjusted values of highest (Max) and lowest (Min) performing variety is depicted along with the arithmetic mean of the evaluated variety set. CoV, Coefficient of variation*.

**Figure 1:**
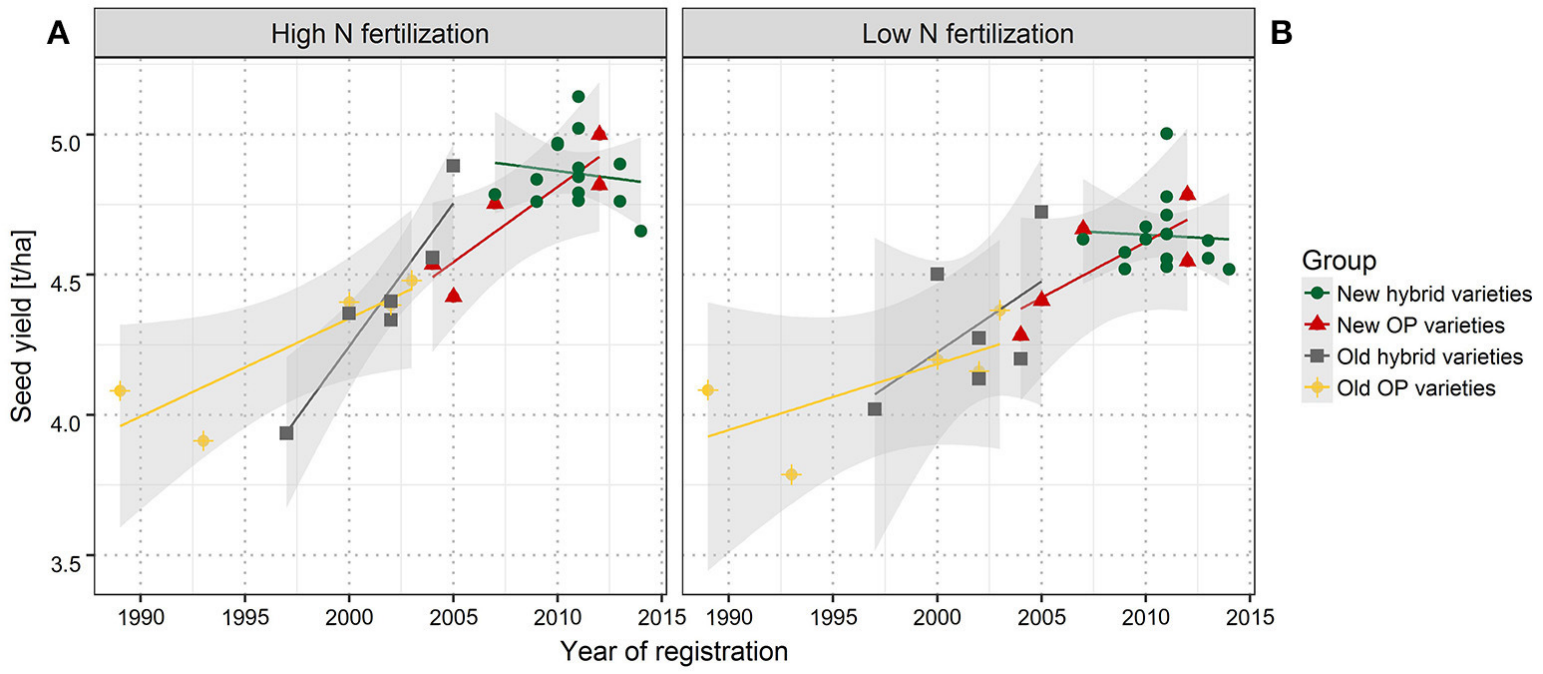
Seed yield (SY) of individual varieties grouped according to the market release. Linear regression of SY with year of registration for high **(A)** and low **(B)** nitrogen fertilization. New hybrid varieties (green dots), new OP varieties (red triangles), old hybrid varieties (gray squares), and old OP varieties (yellow diamonds). Least significant difference of 0.185 (HN) and 0.184 (LN) was estimated on the 5% error level. Gray shaded area indicates the confidence interval.

**Figure 2:**
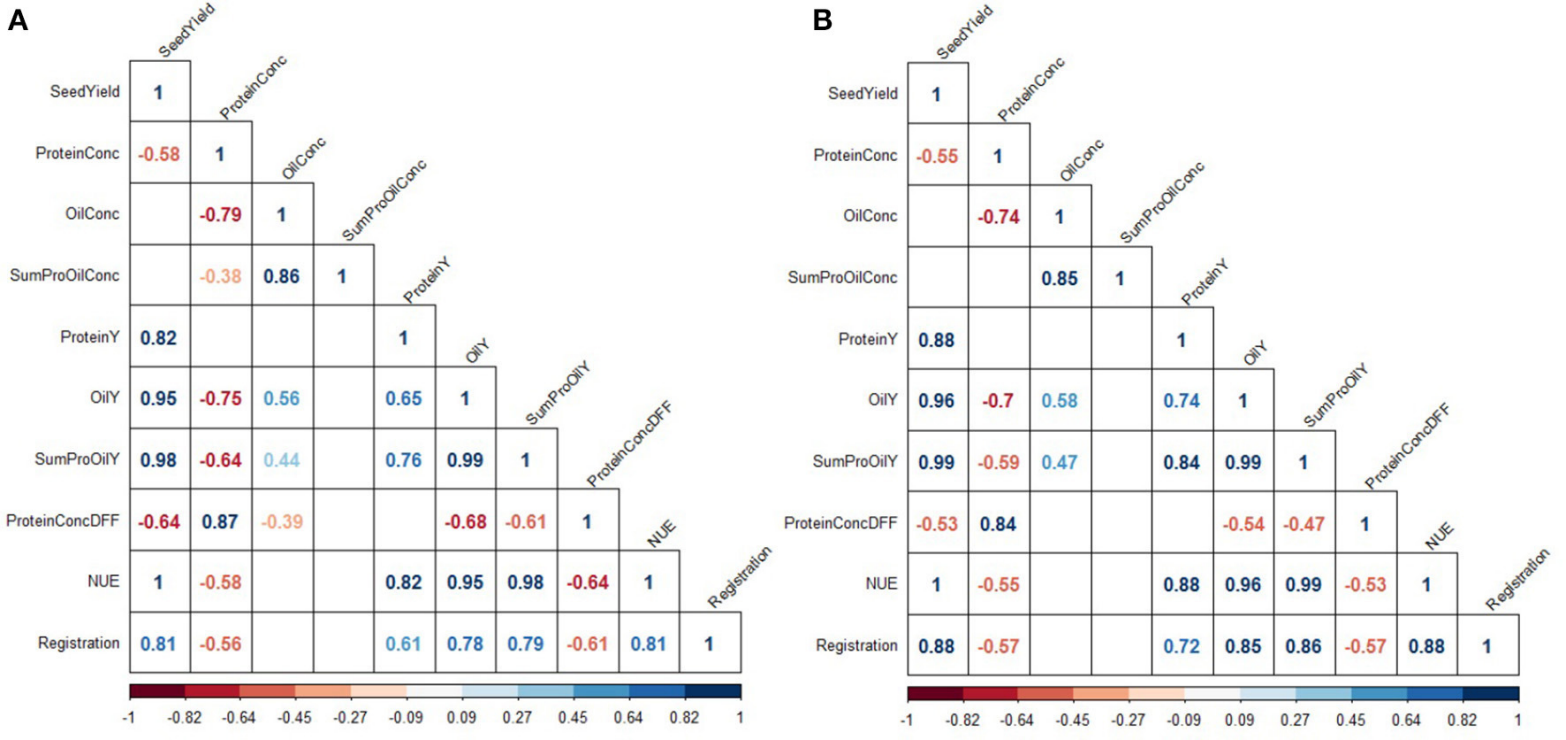
Inter-trait phenotypic correlations at low **(A)** and high **(B)** nitrogen fertilization. Colors indicate the strength of correlations. Only correlations significant at a confidence level of 95% are depicted.

Across the entire set of tested varieties, correlation between SY and the year of registration was *r* = 0.81 for LN and *r* = 0.88 for HN, reflecting a strong breeding progress over the period of observation (Figures 1A,B, 2). This is further underlined by a very high value for broad sense heritability in the investigated variety set (*h*^2^ = 0.92, Table 4). The annual yield progress was ~35 kg ha^−1^ year^−1^ at LN and 45 kg ha^−1^ year^−1^ at HN.

**Table 4:** Analysis of variance and broad sense heritability for investigated traits.

**Trait**	**Environment**	**Variety**	**NFL**	**Variety ×**	***h*^2^**
				**NFL**	
Seed Yield (t/ha)	Mean 2015	^***^	.		
	Mean 2016	^***^			
	Mean	^***^	^*^	.	0.92
ProteinConc (%)	Mean 2015	^***^	^**^		
	Mean 2016	^***^	^***^	.	
	Mean	^***^	^***^	.	0.96
OilConc (%)	Mean 2015	^***^	^**^		
	Mean 2016	^***^	^***^	^*^	
	Mean	^***^	^***^		0.97
ProteinY (t/ha)	Mean 2015	^***^	^*^	^*^	
	Mean 2016	^***^	^***^	.	
	Mean	^***^	^***^	^**^	0.86
OilY (t/ha)	Mean 2015	^***^			
	Mean 2016	^***^			
	Mean	^***^			0.94
ProteinConcDFF (%)	Mean 2015	^***^	^**^	.	
	Mean 2016	^***^	^***^	.	
	Mean	^***^	^***^	.	0.96

*NFL, Nitrogen fertilization level. Level of significance is indicated by . for p < 0.1*,

* *p < 0.01*,

** p < 0.005 and

*** *p < 0.001*.

If the investigated set is split into groups of hybrid and OP varieties and in older and modern varieties, it becomes obvious that according to the respective arithmetic means, modern hybrid varieties outperform old hybrid varieties as modern OP varieties outperform old OP varieties in all the 10 environments and in both the NFL. There is just one exception at HN in ROS16, where the arithmetic mean of older OP varieties is marginally higher than in modern OP varieties. As indicated in Figure 1, at HN, Pearson coefficient of correlation between SY and year of registration was *r* = 0.89 for new OP varieties, *r* = 0.87 for old OP varieties, and *r* = 0.94 for old hybrids (all significant on the 5% error level). In contrast, the correlations at LN of *r* = 0.76 for new OP varieties, *r* = 0.67 for old OP varieties, and *r* = 0.55 for old hybrids are not significant and lower, indicating a weaker association than at HN. Within the group of modern hybrids, no significant correlation between SY and year of registration could be determined either at HN or at LN.

**Figure 3:**
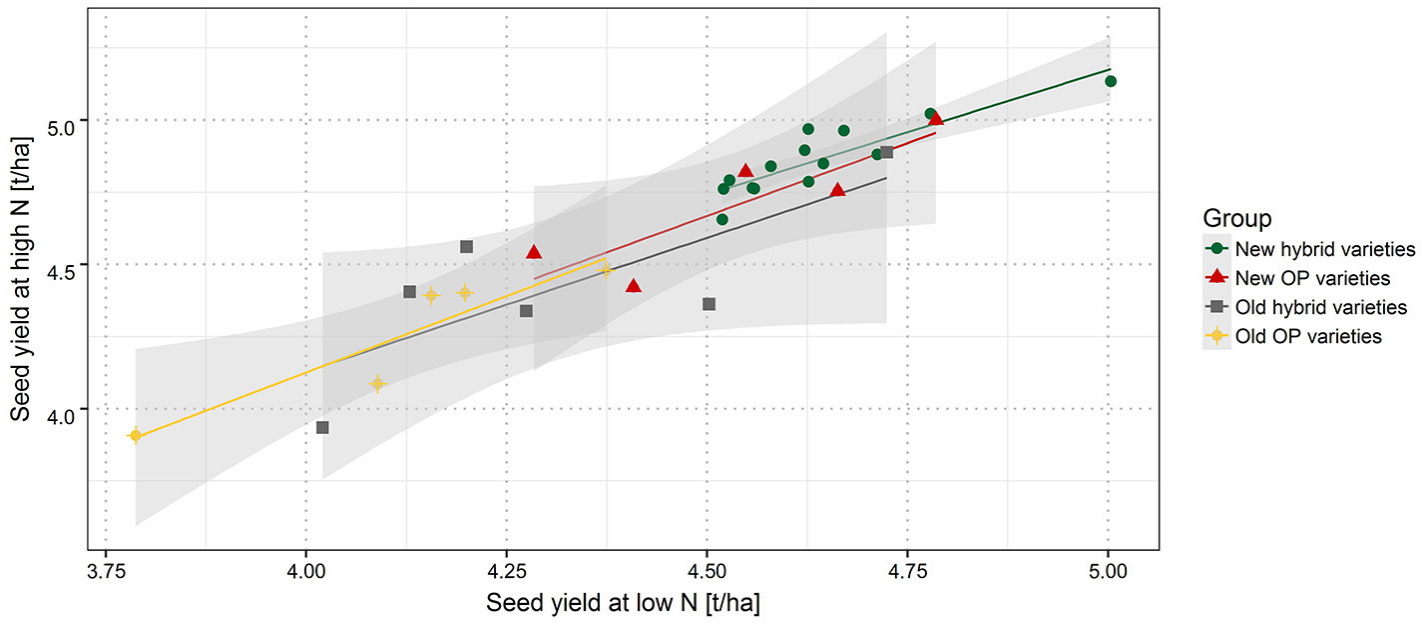
Correlation between SY at high and low nitrogen fertilization. New hybrid varieties (green dots), new OP varieties (red triangles), old hybrid varieties (gray squares), and old OP varieties (yellow diamonds). Gray shaded area indicates the confidence interval.

**Figure 4:**
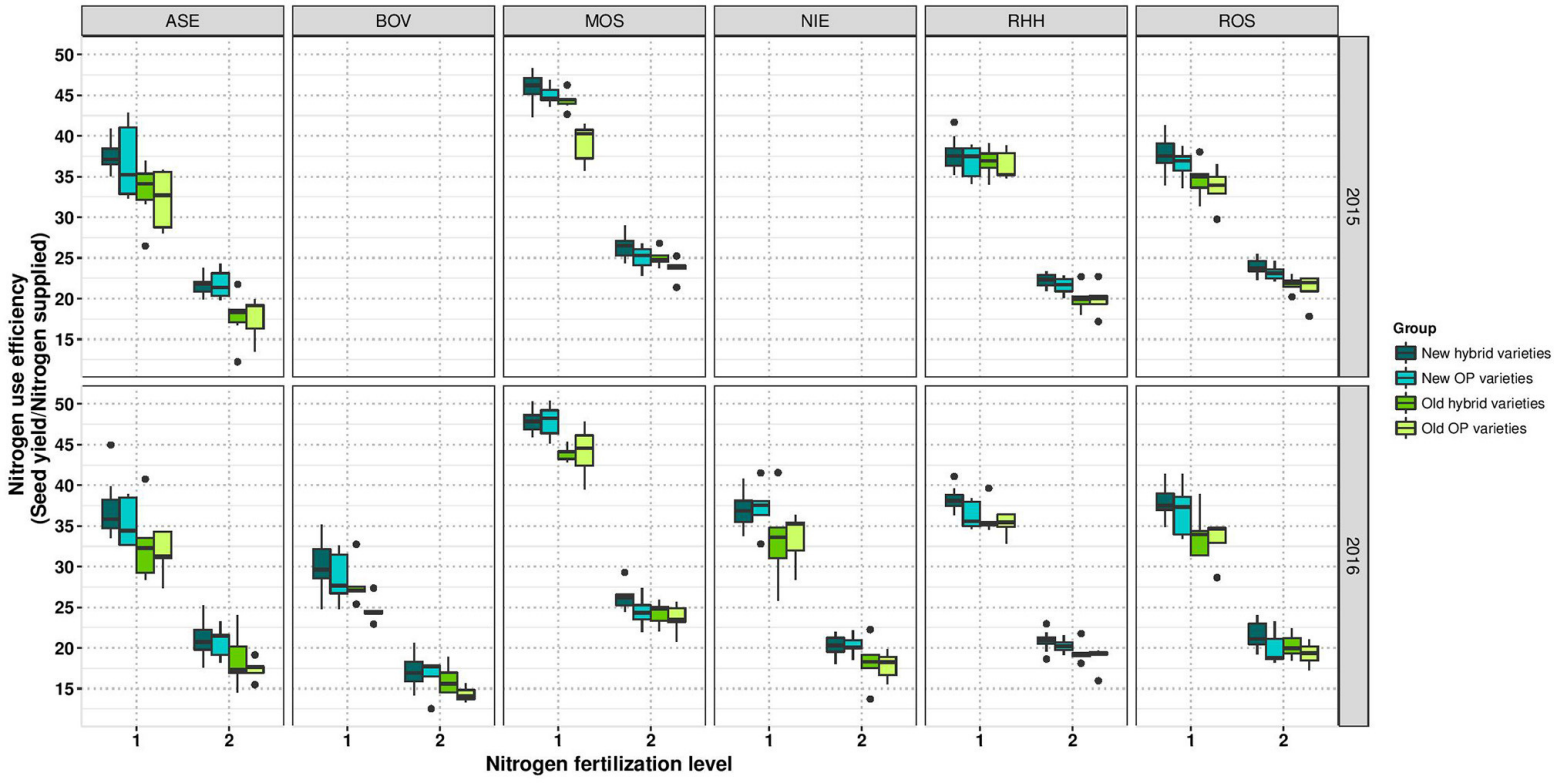
Boxplots for nitrogen use efficiency according to variety groups in individual environments. Data for experimental year 2014–2015 are depicted above and for experimental year 2015–2016 below. Windows include locations, namely Asendorf (ASE), Bovenau (BOV), Moosburg (MOS), Nienstädt (NIE), Rauischholzhausen (RHH), and Rosenthal (ROS). Within each window, nitrogen fertilization level is indicated as 1 for low nitrogen fertilization and 2 for high nitrogen fertilization.

Within the group of old hybrids, it was noticeable that two varieties showed a higher yield of 0.09 t ha^−1^ and 0.14 ha^−1^ under LN than under HN (Figure 3). However, it has to be mentioned that this is only a relative performance. On an absolute level, these varieties are only average or second lowest yielding, respectively. Interestingly, the lower-yielding variety appears to have an extreme above-average lodging score (Supplementary Figure 2). Calculations of NUE, expressed as SY per unit of applied nitrogen fertilizer reveal (i) a much higher NUE at LN than at HN and (ii) a gradient of NUE from modern hybrids over modern OP varieties and old hybrids to old OP varieties (Figure 4).

### Oil

**Figure 5:**
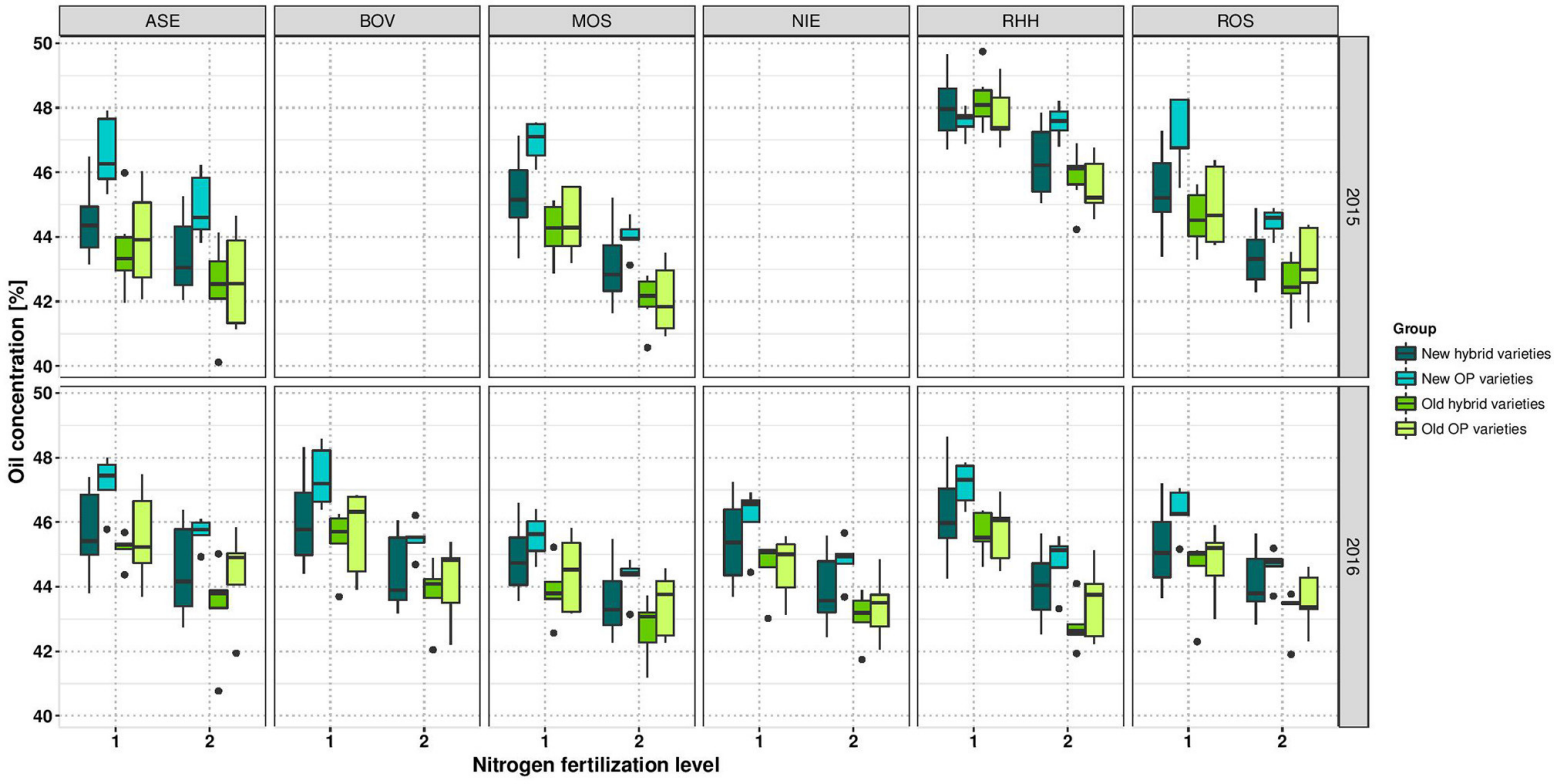
Boxplots for oil concentration according to variety groups in individual environments. Data for experimental year 2014–2015 are depicted above and for experimental year 2015–2016 below. Windows include locations, namely Asendorf (ASE), Bovenau (BOV), Moosburg (MOS), Nienstädt (NIE), Rauischholzhausen (RHH), and Rosenthal (ROS). Within each window, nitrogen fertilization level is indicated as 1 for low nitrogen fertilization and 2 for high nitrogen fertilization.

The ANOVA indicated a significant effect of NFL on OilConc (Table 4). In all environments, OilConc was expectedly higher in LN than in HN treatment. On average, it was 45.64 and 43.96% at LN and HN, respectively, across the entire study, and was almost constant between the investigated years (Table 3). OilConc was found to be very heritable (*h*^2^ = 0.97). A highly significant variety effect was observed in all environments (Supplementary Table 4). With the exception of RHH15 at LN, all individual environments were very high and significantly correlation with each other, ranging between *r* = 0.64 and *r* = 0.93 (Supplementary Figure 3). Furthermore, in RHH15, and BOV16, significant G × N interactions were detected. The correlation between the year of registration and OilConc was only *r* = 0.28 at LN and *r* = 0.30 at HN and not significant. Figure 5 indicates that modern varieties, especially the OP varieties, clearly outperform the older groups at both NFL (but not at LN in RHH15).

Furthermore, the results show that LN fertilization was sufficient to produce the same amount of oil than produced through HN. Since, OilY is the product of SY and OilConc, and both traits showed a contrary response to N fertilization, OilY is the same at both NFL (2.04 t ha^−1^ at LN and HN). The highest OilY difference was observed with 210 kg ha^−1^ at ROS15 (Supplementary Table 3). At both NFL, results have proven that modern varieties require less N fertilization to achieve the same OilY compared to varieties registered in olden times (Supplementary Figure 4). As correlations in Figure 2 illustrated, the OilY is much more determined by SY (LN *r* = 0.95; HN *r* = 0.96) than by oil concentration (LN *r* = 0.56; HN *r* = 0.58). Moreover, correlations of OilY between environments at LN and HN are in most cases above a correlation of *r* = 0.5 and significant, except for RHH15 at LN (Supplementary Figures 5A,B).

### Protein

ProteinConc was significantly affected by variety and NFL across the entire study and at each of the 2 years (Table 4). At LN, ProteinConc was 15.43% at LN, and 90% of the level achieved at HN (17.22%). Also, in each single experiment, the variety and NFL significantly affected ProteinConc, and only in RHH15 and BOV16, G × N interactions were observable (as for OilConc). In summary, Pearson coefficient of correlation between SY and ProteinY of *r* = 0.82 for LN and *r* = 0.88 for HN revealed that latter was through the overwhelming part determined by SY. No significant relationship was found between ProteinConc and ProteinY (Figure 2), indicating that SY was the relevant parameter for N extraction and removal from the field. For experimental year 2015–2016, ProteinConc at LN was with 15.73% higher compared to the first year's trails (14.97%). The ProteinY at LN was with 0.69 t ha^−1^, just 86% of the ProteinY at HN (0.80 t ha^−1^). ProteinConc and ProteinY were the traits with the strongest alteration due to divergent NFL, stronger than oil-related traits (Table 3). As indicated in Figure 2, across the entire set in both NFL, a negative relationship between the year of registration (LN *r* = −0.56; HN *r* = −0.57) was observed. In addition, ProteinConc had an exceptional strong negative relationship with OilConc at LN (*r* = −0.79) and HN (*r* = −0.74). Except for RHH15 at LN protein concentration show a medium to high correlation between environments (Supplementary Figure 6). In contrast, for ProteinY also low correlations between random environments were observed (Supplementary Figure 7).

**Figure 6:**
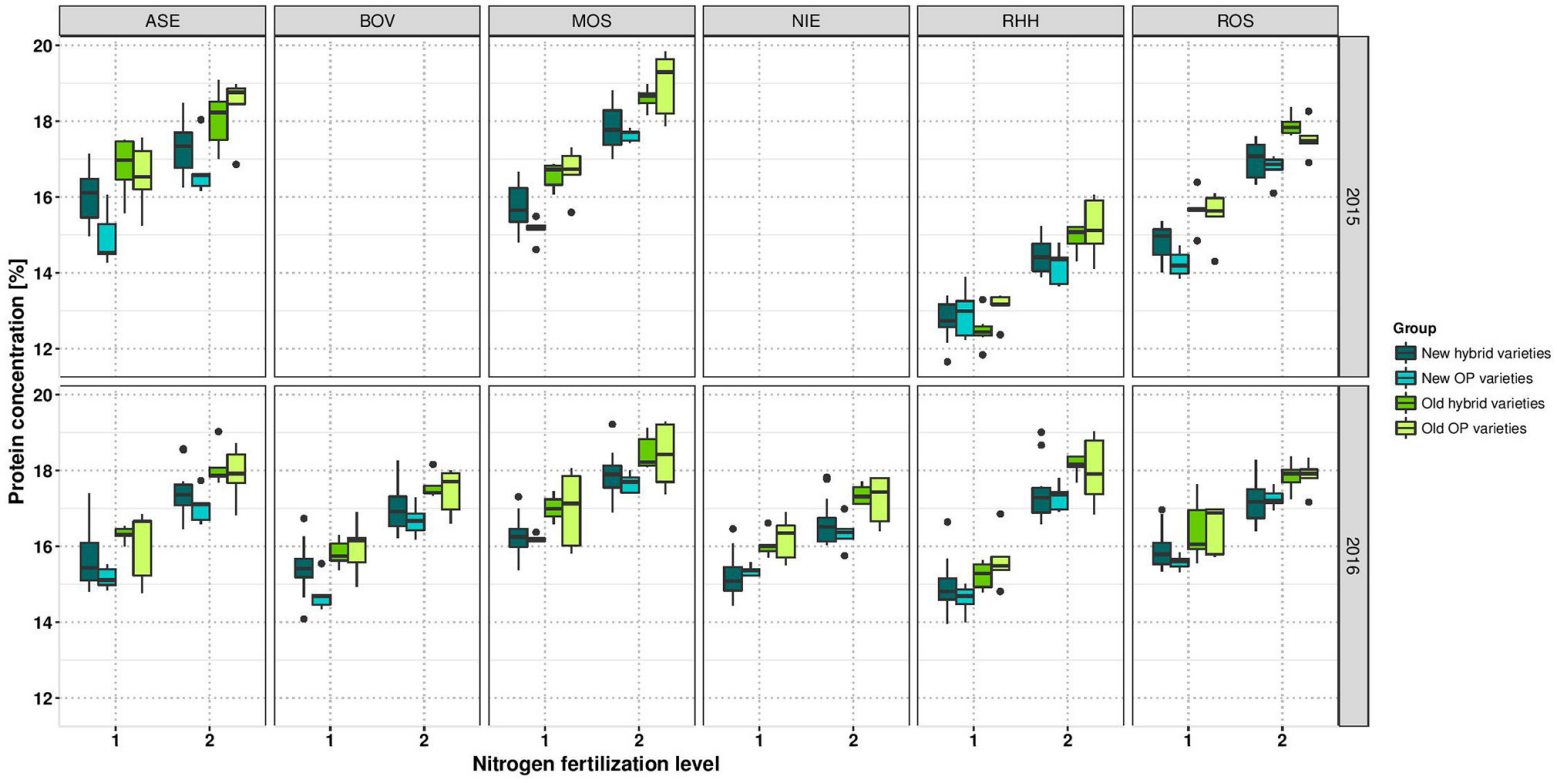
Boxplots for protein concentration according to variety groups in individual environments. Data for experimental year 2014–2015 are depicted above and for experimental year 2015–2016 below. Windows include locations, namely Asendorf (ASE), Bovenau (BOV), Moosburg (MOS), Nienstädt (NIE), Rauischholzhausen (RHH), and Rosenthal (ROS). Within each window, nitrogen fertilization level is indicated as 1 for low nitrogen fertilization and 2 for high nitrogen fertilization.

If the diversity set is separated into the four variety groups, it become obvious that the oldest varieties, in most cases the old OP varieties, have the highest protein concentration. On the contrary, modern varieties, predominantly modern OP varieties, have the lowest protein concentration (Figure 6). Old OP varieties (LN *r* = −0.61; HN *r* = −0.47) and modern OP varieties (LN *r* = −0.50; HN *r* = +0.64) have weaker correlation between OilConc and ProteinConc, while old hybrids (LN *r* = −0.81; HN *r* = −0.88) and modern hybrids (LN *r* = −0.81; HN *r* = −0.76) have the strongest negative correlation.

## Discussion

### Measurement of breeding progress in a complex interacting growth system

NUE is the final outcome of a complex cropping system with Genotype × Environment × Management (G × E × M) interactions (Dresbøll and Thorup-Kristensen, 2014; Thorup-Kristensen and Kirkegaard, 2016). While environmental conditions such as rainfall, temperature, radiation, and others cannot be influenced, the management of crop rotation and decision on soil tillage, as well as dosage and timing of sowing, fertilization, and responsive use of plant protection agents can be modified by farmers. In addition, since the effect of the preceding crop on the following crop has to be taken into account, the realized NUE has to be measured within the cropping system level as the relevant benchmark (Dresbøll and Thorup-Kristensen, 2014). Within this system, the use of most appropriate genotypes is an important question. In this regard, the aim of our study was to understand the relevance of the genotype of oilseed rape and how breeding influences the progress of its NUE. Precise extraction of genetic contribution for alteration of NUE is extremely challenging due to a lack of comparable environmental conditions. This is not only because SY is determined over a lengthy maturation period, but also because numerous environmental factors and management decisions, including N fertilizer applications, influence various components of NUE throughout the growing season. Therefore, investigation of breeding progress requires testing of varieties from different periods of registration under exactly the same conditions, however conditions always differ between individual experiments or replicates. In this study, the experiments were conducted after wheat or barley and crop management at all locations was oriented on a commonly used, relatively intensive Northern European oilseed rape production system.

The results show that the locations chosen for this study were subject to contrasting environmental influences. For example, in BOV16, the yields were extremely low due to a combination of pest and disease pressure at a historic low level (C. Algermissen, personal communication). On the contrary, MOS15 and MOS16 were characterized by favorable growth conditions, resulting in significant above-average yield levels.

Despite the divergent environmental influences and complex interactions of NUE, due to its highly quantitative inheritance, the high heritability determined in our study (*h*^2^ = 0.92 for SY and *h*^2^ = 0.94 for OilY) indicates that the enhancement of NUE can be achieved through genetic improvement. Thus, over a long period of almost 25 years, breeding was revealed to be a successful strategy, even when gains from year to year are rather small. This finding is in line with modeling studies by Dresbøll and Thorup-Kristensen (2014).

Several studies have described a genotype by nitrogen (G × N) interaction for several major crops (Foulkes et al., 2009; Gaju et al., 2014). In this study the overall picture (Figure 3) suggests that there is a very strong correlation between HN and LN. Also at most locations the correlation between HN and LN is higher than correlations between locations. This suggests that soil, weather and climate conditions often had a stronger effect than variety specific reaction to NFL. For example, the best performing variety at HN in MOS16 performs only on average in ASE16 and vice versa (Supplementary Figure 1). This finding is in line with previous studies on oilseed rape in France, where most quantitative trait loci were constitutive under LN and HN fertilization for yield parameters but not between environments (Bouchet et al., 2014, 2016).

### Yield increase drives NUE

In this work, a collection of winter type oilseed rape varieties with major market importance in Germany and neighboring Northern-European countries was investigated. In contrast to a previous study on NUE in oilseed rape (Stahl et al., 2016), where genetic diversity for NUE was investigated in a broad collection of winter type oilseed rape associations, this study was conducted only on elite varieties that were registered between 1989 and 2014. Thus, the results are not biased through tipping points in breeding history of oilseed rape, as the introduction of zero erucic acid and low glucosinolate content varieties (Downey et al., 1969). In contrast to an earlier study (Kessel et al., 2012) investigating the breeding progress before 1999, the present study focuses on a very recent time window. The fact that varieties such as Express and Lirajet, labeled as modern varieties in Kessel et al. (2012) but are by far the oldest varieties in our study, is indicative of the sliding window in breeding history. Finally, since hybrid varieties have become important and meanwhile dominate oilseed rape production in Northern Europe, a reassessment of breeding progress was overdue. This issue is especially relevant due to several indications for a heterosis effect on yield, which is often pronounced particularly under limited N conditions (Gehringer et al., 2007; Koeslin-Findeklee et al., 2014; Wang et al., 2016).

The ultimate yardstick for mitigating N losses and to create a most N sustainable oilseed rape production is the amount of nitrogen, which is added to the cultivation system required to harvest one unit seed or oil (and protein). The same relationship is expressed by the reciprocal relationship, which is the widely used definition of NUE, as the SY over supplied N (Moll et al., 1982; Good et al., 2004). The above presented results provide evidence for a tremendously successful increase in SY and OilY, and are in the overall picture with many previously described data for oilseed rape (Kessel et al., 2012; Koeslin-Findeklee et al., 2014), wheat (Austin et al., 1980; Fischer and Edmeades, 2010; Cormier et al., 2013; Laidig et al., 2017a), maize (Tollenaar, 1989), rye (Laidig et al., 2017b); rice (Zhu et al., 2016), and triticale (Losert et al., 2017). Therefore, the sometimes appearing hypothesis that cultivation of older varieties could be beneficial for better nutrient use, since they might have comparative advantages in adaptation to low input systems, can unambiguously be rejected.

Since modern high-yielding varieties achieve much higher yields (SY, OilY, and ProteinY), while N inputs and management were the same as those for the old low-yielding varieties, it can be concluded that N losses are significantly lower in modern varieties. In conclusion, the amount of N fertilizer required to produce 1 ton of oilseed rape has dramatically declined within the last 25 years (Supplementary Figure 4). Therefore, our results support the statement of Burney et al. (2010) that increasing the yield is a highly effective instrument to reduce the negative effects on the environment. This is not only because of a higher yield to fertilizer ratio but also due to a lower cultivation area that is required to produce the same amount of commodities. Thus, increasing yields reduces indirect land-use change due to oilseed rape production (Don et al., 2012).

In our study, a strong increase in SY and NUE was observed for both HN and LN, with a high correlation between both the treatments. Bouchet et al. (2014) also found only small G × N interactions when NFL differs by 80–90 kg N ha^−1^. Nevertheless, the stronger correlation between the year of registration and SY indicates that the progress was slightly more pronounced at HN. Similarly, the majority of studies (e.g., Brancourt-Hulmel et al., 2005; Brisson et al., 2010) pointed out that progress in SY was higher at HN than at LN. Over the last few decades, farmers became used to rather high amounts of N fertilization, while breeders tried to treat their selection environments in a manner similar to common farming practice in order to select the genotypes for the target environment. This suggests that the selection for HN conditions was probably a direct selection, while selection for LN was rather indirect, and thus not quite so effective as selection under HN conditions. The results of our study should encourage breeders to select their varieties directly at reduced NFL, in order to speed up the breeding progress for low N inputs.

The breeding progress described here is still an ongoing process. Although, Figure 1 shows a complete absence or even a negative slope for modern hybrids, unpublished data from varieties released to the market after 2014 helps to conclude that breeding for high yielding and more efficient varieties has not come to an end and is rather still an ongoing process.

### Seed quality in light of efficient nitrogen use

The analysis of the breeding success depends not only on yield quantities but also on the changes in the specific seed quality composition. The strong positive correlation between the year of registration and OilY can be explained by the fact that oil was an economically relevant component, with farmers in Germany paid a premium for a high-oil crop, hence breeders have selected genotypes high in oil (Abbadi and Leckband, 2011). OilY increased in an over-proportional manner along with a simultaneous increase in SY. Although, the new OP varieties investigated in this study are, on average, not higher yielding than new hybrids, we observed that they outperformed all other varieties in terms of OilConc under both HN and LN (Figure 5).

On the contrary, the improvement of OilConc correlated with a decrease in ProteinConc (Figure 2). The negative correlation of −0.79 for LN and −0.74 for HN is consistent with studies reported earlier (Bouchet et al., 2014, 2016; Nyikako et al., 2014). Since, oil and protein synthesis are supposed to rely on the same carbon sources, and protein synthesis is preferentially enhanced with increasing N availability, OilConc declines with increased N fertilization (Rathke et al., 2006; Zhao et al., 2006). However, ANOVA (Table 4) illustrates that the effect of NFL is more pronounced on ProteinConc than on OilConc.

From the perspective of resource efficiency, a high ProteinY is desired, since it determines the proportion of N that is captured in the harvested plant organs (as the sink), removed from the field, and thus, protected from losses. Although a strong reduction of ProteinConc is evident in groups of modern varieties, the proportion of N removed from the field compared to proportion invested has improved significantly by overcompensation of a SY-driven enhancement of ProteinY. This finding is in agreement with previously published results from Koeslin-Findeklee et al. (2014).

While OilY and ProteinY are positively correlated to each other (Figure 2), since both are predominantly explained by the common factor SY, the improvement of ProteinY through ProteinConc is hampered by the trade-off. Nevertheless, although the notion that the strong negative correlation between OilConc and ProteinConc makes it impossible to increase OilY to N fertilization ratio through enhanced ProteinConc, it does not necessarily mean that breeders are unable to increase the sink capacity due to selection of quality traits. A selection of higher ProteinConcDFF can be a promising strategy to increase the amount of N stored and harvested in seeds without neglecting the achievements in high OilConc (Potter et al., 2016). In this case, the correlation is weakly negatively correlated to OilConc (*r* = −0.39 of ProteinConcDFF vs. *r* = −0.79 of ProteinConc at LN, Figure 2). However, this strategy would require that the protein content in the meal is included as a couple product in ecological footprint calculations and furthermore receives the necessary economic attention to justify breeders' attempts to select genotypes superior in this trait.

### Scope for reduced fertilizer inputs without drastic yield penalties

Choosing NFL that are suitable to phenotype responses to N is a non-trivial question for farming practices, selection decisions by breeders, and for experimental setup in crop research. Han et al. (2015) reviewed that phenotypic data collected under a severe N stress are not comparable to mild N stress. In some studies (Kessel et al., 2012; Miersch et al., 2016), N stress was maximized by zero N treatment in order to observe genotypes' response to provoked severe stress. On the other hand, zero N is not a realistic scenario for future oilseed rape production. Even if one considers a reduction in the maximum allowance of N application under upcoming stricter environmental policies, a certain stock application of N is always inevitable to achieve crop yields that allow rentable crop cultivation. Furthermore, simply for the reason, that soil exploitation has to be avoided N fertilization will always be essential to re-deliver the removed N in sustainable farming practice. For this reason, the application of 120 kg N ha^−1^ instead of zero N is much closer to reality. The low NFL represents a below-average application rate that is clearly below the maximum NFL that will legally be applicable according to future fertilizer ordinance. The high NFL of 220 kg ha^−1^ was oriented on today's common farming practice (Rathke et al., 2006).

The average SY difference between both NFL of 180 kg ha^−1^ found in our study was surprisingly low. Even the highest SY difference of 690 kg ha^−1^ is comparably small considering the delta of 100 kg N fertilizer between both the treatments. Thus, in our study, the additional fertilizer application did not result in higher yields but rather contributed to a higher N balance surplus. For the first experimental year, one might speculate that observation is explainable due to equal fertilization dosage at the first application date in conjunction with a potentially low availability of N after the second application due to limitations in rainfall in central Germany. Although, the interaction between water limitation and nitrogen uptake (Albert et al., 2012; Sadras and Lawson, 2013) is a reasonable explanation, this logic does not hold true for MOS15, where high rainfalls were observed during spring, and not for the second experimental year, where fertilizer application was reduced at both application dates. Therefore, we have to conclude in all environments of this study that N dosage of much < 220 kg N ha^−1^ is sufficient to produce yield levels that are usually achieved in agricultural farming practice. Since, there is a linear relationship between N fertilizer inputs and energy inputs, the reduction of NFL not only provides an advantageous effect on the mitigation of greenhouse gas emissions but also provides a drastic profit for energy balance of oilseed rape production.

Even if our results cannot be generalized and might not be applicable to all future growth scenarios, our findings that the yield difference between both NFL was very low, in 10 independent experiments conducted across Germany in 2 years, suggest a remarkable potential for reduction in N fertilization without dramatic yield penalties However, this is depending on the weather conditions, which are not known in advance. The fact that farmers have to make their fertilizing decisions in absence of knowledge about further growth conditions makes it difficult to precisely adjust NFL to the real demand (Henke et al., 2007). For further field-based research experiments with contrasting NFL, we suggest to lower the N fertilization in both treatments and agree with Miersch et al. (2016), who suggested to use a delta of at least 100 kg N ha^−1^ between NFL.

## Conclusion

The study was designed to evaluate the effect of plant breeding on NUE in the past decades and the adjustments in selection required to address future sustainability goals. The experimental data provide strong evidence that direct selection for SY and seed oil concentration leads not only to an enormous (oil) yield gain in highly fertilized environments, but also to a selection of genotypes with superior performance in low N-input cultivation systems. Thus, genetic improvement increases NUE in oilseed rape and reduces the reliance on fertilizer inputs, as already suggested for other crops (Hawkesford, 2014). From this perspective, we concur with Burney et al. (2010) that yield improvement should play a predominant role in strategies toward GHG emission mitigation and enhancement of sustainability of crop production.

The surprisingly low yield gap between high and low nitrogen fertilization provides promising hints toward further N fertilizer-saving potential. However, transfer of these results into knowledge-based farming practices remains challenging. For a more precise and directed selection of even more efficient varieties, a better understanding of G × E × M interaction und physiological determinants of NUE are essential tasks for future research.

## Author contributions

RS, BW, and AS conceived the research; AS and MP performed the experiments, AS and MF performed data analysis; AS and RS wrote the manuscript.

### Conflict of interest statement

The authors declare that the research was conducted in the absence of any commercial or financial relationships that could be construed as a potential conflict of interest.

## Abbreviations

GHG: Greenhouse gas
HN: High nitrogen
LN: Low nitrogen
N: Nitrogen
N_min_: Soil mineral nitrogen
NFL: Nitrogen fertilization level
NUE: Nitrogen use efficiency (as Seed yield over N supplied)
OilConc: Seed oil concentration (at 91% dry matter content)
OilY: Oil yield
OP: Open pollinated
ProteinConc: Seed protein concentration (at 91% dry matter content)
ProteinConc DFF: ProteinConc in defatted fraction (at 91% dry matter content)
ProteinY: Protein yield
SumProOilConc: Sum of protein and oil concentration (at 91% dry matter content)
SumProOilY: Sum of protein and oil yield
SDH: Semi dwarf hybrid
SY: Seed yield (at 91% dry matter content).

## References

[B1] Abbadi A. Leckband G. (2011). Rapeseed breeding for oil content, quality, and sustainability. Eur. J. Lipid Sci. Technol. 113, 1198–1206. 10.1002/ejlt.201100063

[B2] Albert B. Le Cahérec F. Niogret M.-F. Faes P. Avice J.-C. Leport L.. (2012). Nitrogen availability impacts oilseed rape (*Brassica napus* L.) plant water status and proline production efficiency under water-limited conditions. Planta 236, 659–676. 10.1007/s00425-012-1636-8 22526495 PMC3404282

[B3] Austin R. B. Bingham J. Blackwell R. D. Evans L. T. Ford M. A. Morgan C. L. (1980). Genetic improvements in winter wheat yields since 1900 and associated physiological changes. J. Agric. Sci. 94, 675 10.1017/S0021859600028665

[B4] Bates D. Maechler M. Bolker B. Walker S. (2015). Fitting linear mixed-effects models using lme4. J. Stat. Softw. 67, 1–48. 10.18637/jss.v067.i01

[B5] Bouchet A.-S. Laperche A. Bissuel-Belaygue C. Baron C. Morice J. Rousseau-Gueutin M.. (2016). Genetic basis of nitrogen use efficiency and yield stability across environments in winter rapeseed. BMC Genet. 17:131. 10.1186/s12863-016-0432-z 27628849 PMC5024496

[B6] Bouchet A.-S. Nesi N. Bissuel C. Bregeon M. Lariepe A. Navier H. (2014). Genetic control of yield and yield components in winter oilseed rape (*Brassica napus* L.) grown under nitrogen limitation. Euphytica 199, 183–205. 10.1007/s10681-014-1130-4

[B7] Brancourt-Hulmel M. Heumez E. Pluchard P. Beghin D. Depatureaux C. Giraud A.. (2005). Indirect versus direct selection of winter wheat for low-input or high-input levels. Crop Sci. 45, 1427–1431. 10.2135/cropsci2003.034325172085

[B8] Brisson N. Gate P. Gouache D. Charmet G. Oury F.-X. Huard F. (2010). Why are wheat yields stagnating in Europe? A comprehensive data analysis for France. Field Crops Res. 119, 201–212. 10.1016/j.fcr.2010.07.012

[B9] Burney J. A. Davis S. J. Lobell D. B. (2010). Greenhouse gas mitigation by agricultural intensification. Proc. Natl. Acad. Sci. U.S.A. 107, 12052–12057. 10.1073/pnas.0914216107 20551223 PMC2900707

[B10] Cormier F. Faure S. Dubreuil P. Heumez E. Beauchene K. Lafarge S.. (2013). A multi-environmental study of recent breeding progress on nitrogen use efficiency in wheat (*Triticum aestivum L*.). Theor. Appl. Genet. 126, 3035–3048. 10.1007/s00122-013-2191-9 24057081

[B11] De Mendiburu F. Simon R. (2015). Agricolae - ten years of an open source statistical tool for experiments in breeding, agriculture and biology. PeerJ PrePrints 3:e1404v1. 10.7287/peerj.preprints.1404v1

[B12] Don A. Osborne B. Hastings A. Skiba U. Carter M. S. Drewer J. (2012). Land-use change to bioenergy production in Europe: implications for the greenhouse gas balance and soil carbon. Glob. Change Biol. Bioenergy 4, 372–391. 10.1111/j.1757-1707.2011.01116.x

[B13] Downey R. K. Craig B. M. Youngs C. G. (1969). Breeding rapeseed for oil and meal quality. J. Am. Oil Chem. Soc. 46, 121–123. 10.1007/BF02635712

[B14] Dresbøll D. B. Thorup-Kristensen K. (2014). Will breeding for nitrogen use efficient crops lead to nitrogen use efficient cropping systems? A simulation study of G × E × M interactions. Euphytica 199, 97–117. 10.1007/s10681-014-1199-9

[B15] Fischer R. A. Byerlee D. Edmeades G. O. (2014). Crop Yields and Global Food Security: Will Yield Increase Continue to Feed the World? ACIAR Monograph No. 158. Australian Centre for International Agricultural Research, Canberra, ACT.

[B16] Fischer R. A. Edmeades G. O. (2010). Breeding and cereal yield progress. Crop Sci. 50, S-85–S-98. 10.2135/cropsci2009.10.0564

[B17] Fleddermann M. Fechner A. Rossler A. Bahr M. Pastor A. Liebert F.. (2013). Nutritional evaluation of rapeseed protein compared to soy protein for quality, plasma amino acids, and nitrogen balance—a randomized cross-over intervention study in humans. Clin. Nutr. 32, 519–526. 10.1016/j.clnu.2012.11.005 23260747

[B18] Foulkes M. J. Hawkesford M. J. Barraclough P. B. Holdsworth M. J. Kerr S. Kightley S. (2009). Identifying traits to improve the nitrogen economy of wheat: recent advances and future prospects. Field Crops Res. 114, 329–342. 10.1016/j.fcr.2009.09.005

[B19] Gaju O. Allard V. Martre P. Le Gouis J. Moreau D. Bogard M. (2014). Nitrogen partitioning and remobilization in relation to leaf senescence, grain yield and grain nitrogen concentration in wheat cultivars. Field Crops Res. 155, 213–223. 10.1016/j.fcr.2013.09.003 PMC7182295 32549650

[B20] Galloway J. N. Leach A. M. Bleeker A. Erisman J. W. (2013). A chronology of human understanding of the nitrogen cycle. Philos. Trans. R. Soc. Lond. B Biol. Sci. 368:20130120. 10.1098/rstb.2013.0120 23713118 PMC3682740

[B21] Gehringer A. Snowdon R. Spiller T. Basunanda P. Friedt W. (2007). New oilseed rape (*Brassica napus*) hybrids with high levels of heterosis for seed yield under nutrient-poor conditions. Breed. Sci. 57, 315–320. 10.1270/jsbbs.57.315

[B22] Good A. G. Shrawat A. K. Muench D. G. (2004). Can less yield more? Is reducing nutrient input into the environment compatible with maintaining crop production? Trends Plant Sci. 9, 597–605. 10.1016/j.tplants.2004.10.008 15564127

[B23] Han M. Okamoto M. Beatty P. H. Rothstein S. J. Good A. G. (2015). The genetics of nitrogen use efficiency in crop plants. Annu. Rev. Genet. 49, 269–289. 10.1146/annurev-genet-112414-055037 26421509

[B24] Hawkesford M. J. (2014). Reducing the reliance on nitrogen fertilizer for wheat production. J. Cereal Sci. 59, 276–283. 10.1016/j.jcs.2013.12.001 24882935 PMC4026125

[B25] Henke J. Böttcher U. Neukam D. Sieling K. Kage H. (2008). Evaluation of different agronomic strategies to reduce nitrate leaching after winter oilseed rape (*Brassica napus* L.) using a simulation model. Nutr. Cycl. Agroecosyst. 82, 299–314. 10.1007/s10705-008-9192-0

[B26] Henke J. Breustedt G. Sieling K. Kage H. (2007). Impact of uncertainty on the optimum nitrogen fertilization rate and agronomic, ecological and economic factors in an oilseed rape based crop rotation. J. Agric. Sci. 145, 455 10.1017/S0021859607007204

[B27] Hirel B. Le Gouis J. Ney B. Gallais A. (2007). The challenge of improving nitrogen use efficiency in crop plants: towards a more central role for genetic variability and quantitative genetics within integrated approaches. J. Exp. Bot. 58, 2369–2387. 10.1093/jxb/erm097 17556767

[B28] Kant S. Bi Y.-M. Rothstein S. J. (2011). Understanding plant response to nitrogen limitation for the improvement of crop nitrogen use efficiency. J. Exp. Bot. 62, 1499–1509. 10.1093/jxb/erq297 20926552

[B29] Kessel B. Schierholt A. Becker H. C. (2012). Nitrogen use efficiency in a genetically diverse set of winter oilseed rape (*Brassica napus* L.). Crop Sci. 52, 2546–2554. 10.2135/cropsci2012.02.0134

[B30] Koeslin-Findeklee F. Meyer A. Girke A. Beckmann K. Horst W. J. (2014). The superior nitrogen efficiency of winter oilseed rape (*Brassica napus* L.) hybrids is not related to delayed nitrogen starvation-induced leaf senescence. Plant Soil 384, 347–362. 10.1007/s11104-014-2212-8

[B31] Kumar A. Sharma A. Upadhyaya K. C. (2016). Vegetable oil: nutritional and industrial perspective. Curr. Genomics 17, 230–240. 10.2174/1389202917666160202220107 27252590 PMC4869010

[B32] Kuznetsova A. Brockhoff P. B. Christensen R. H. B. (2016). lmerTest: Tests in Linear Mixed Effects Models. R Package version 2.0–30.

[B33] Laidig F. Piepho H. P. Rentel D. Drobek T. Meyer U. Huesken A. (2017a). Breeding progress, environmental variation and correlation of winter wheat yield and quality traits in German official variety trials and on-farm during 1983–2014. Theor. Appl. Genet. 130, 223–245. 10.1007/s00122-016-2810-3 27796431 PMC5215243

[B34] Laidig F. Piepho H. P. Rentel D. Drobek T. Meyer U. Huesken A. (2017b). Breeding progress, variation, and correlation of grain and quality traits in winter rye hybrid and population varieties and national on-farm progress in Germany over 26 years. Theor. Appl. Genet. 130, 981–998. 10.1007/s00122-017-2865-9 28289803 PMC5395587

[B35] Lenth V. R. (2016). Least-squares means: the R package lsmeans. J. Stat. Softw. 69, 1–33. 10.18637/jss.v069.i01

[B36] Liu J. You L. Amini M. Obersteiner M. Herrero M. Zehnder A. J. B.. (2010). A high-resolution assessment on global nitrogen flows in cropland. Proc. Natl. Acad. Sci. U.S.A. 107, 8035–8040. 10.1073/pnas.0913658107 20385803 PMC2867927

[B37] Lopes M. S. Reynolds M. P. Manes Y. Singh R. P. Crossa J. Braun H. J. (2012). Genetic yield gains and changes in associated traits of CIMMYT spring bread wheat in a “historic” set representing 30 years of breeding. Crop Sci. 52, 1123–1131. 10.2135/cropsci2011.09.0467

[B38] Losert D. Maurer H. P. Marulanda J. J. Würschum T. (2017). Phenotypic and genotypic analyses of diversity and breeding progress in European triticale (× Triticosecale Wittmack). Plant Breed. 136, 18–27. 10.1111/pbr.12433

[B39] Miersch S. Gertz A. Breuer F. Schierholt A. Becker H. C. (2016). Influence of the Semi-dwarf growth type on seed yield and agronomic parameters at low and high nitrogen fertilization in winter oilseed rape. Crop Sci. 56, 1573–1585. 10.2135/cropsci2015.09.0554

[B40] Moll R. H. Kamprath E. J. Jackson W. A. (1982). Analysis and interpretation of factors which contribute to efficiency of nitrogen utilization1. Agron. J. 74, 562 10.2134/agronj1982.00021962007400030037x

[B41] Müller N. D. Gerber J. S. Johnston M. Ray D. K. Ramankutty N. Foley J. A. (2012). Closing yield gaps through nutrient and water management. Nature 490, 254–257. 10.1038/nature11420 22932270

[B42] Nyikako J. Schierholt A. Kessel B. Becker H. C. (2014). Genetic variation in nitrogen uptake and utilization efficiency in a segregating DH population of winter oilseed rape. Euphytica 199, 3–11. 10.1007/s10681-014-1201-6

[B43] Potter T. Burton W. Edwards J. Wratten N. Mailer R. Salisbury P. (2016). Assessing progress in breeding to improve grain yield, quality and blackleg (*Leptosphaeria maculans*) resistance in selected Australian canola cultivars (1978-2012). Crop Pasture Sci. 67, 308–316. 10.1071/CP15290

[B44] Rathke G. Behrens T. Diepenbrock W. (2006). Integrated nitrogen management strategies to improve seed yield, oil content and nitrogen efficiency of winter oilseed rape (*Brassica napus* L.): a review. Agric. Ecosyst. Environ. 117, 80–108. 10.1016/j.agee.2006.04.006

[B45] Rathke G.-W. Diepenbrock W. (2006). Energy balance of winter oilseed rape (*Brassica napus* L.) cropping as related to nitrogen supply and preceding crop. Eur. J. Agron. 24, 35–44. 10.1016/j.eja.2005.04.003

[B46] R Core Team (2013). A Language and Environment for Statistical Computing. Vienna: R Foundation for Statistical Computing.

[B47] Reinhardt T.-C. (1992). Entwicklung und Anwendung von Nah-Infrarot-Spektroskopischen Methoden für die Bestimmung von Ol-, Protein-, Glucosinolat-, Feuchte- und Fettsaure- Gehalten in intakter Rapssaat. Ph.D. dissertation. University of Gottingen.

[B48] Rockström J. Steffen W. Noone K. Persson A. Chapin F. S. III. Lambin E. F.. (2009). A safe operating space for humanity. Nature 461, 472–475. 10.1038/461472a 19779433

[B49] Rothstein S. J. (2007). Returning to our roots: making plant biology research relevant to future challenges in agriculture. Plant Cell 19, 2695–2699. 10.1105/tpc.107.053074 17873097 PMC2048712

[B50] Sadras V. O. Lawson C. (2013). Nitrogen and water-use efficiency of Australian wheat varieties released between 1958 and 2007. Eur. J. Agron. 46, 34–41. 10.1016/j.eja.2012.11.008

[B51] Sieling K. Kage H. (2006). N balance as an indicator of N leaching in an oilseed rape – winter wheat – winter barley rotation. Agric. Ecosyst. Environ. 115, 261–269. 10.1016/j.agee.2006.01.011

[B52] Stahl A. Friedt W. Wittkop B. Snowdon R. J. (2016). Complementary diversity for nitrogen uptake and utilisation efficiency reveals broad potential for increased sustainability of oilseed rape production. Plant Soil 400, 245–262. 10.1007/s11104-015-2726-8

[B53] Steffen W. Richardson K. Rockstrom J. Cornell S. E. Fetzer I. Bennett E. M.. (2015). Sustainability. Planetary boundaries: guiding human development on a changing planet. Science 347:1259855. 10.1126/science.1259855 25592418

[B54] Sylvester-Bradley R. Kindred D. R. (2009). Analysing nitrogen responses of cereals to prioritize routes to the improvement of nitrogen use efficiency. J. Exp. Bot. 60, 1939–1951. 10.1093/jxb/erp116 19395389

[B55] Thorup-Kristensen K. Kirkegaard J. (2016). Root system-based limits to agricultural productivity and efficiency: the farming systems context. Ann. Bot. 118, 573–592. 10.1093/aob/mcw122 PMC5055631 27411680

[B56] Tillmann P. Paul C. (1998). The repeatability file—a tool for reducing the sensitivity of near infrared spectroscopy calibrations to moisture variation. J. Near Infrared Spectrosc. 6:61 10.1255/jnirs.122

[B57] Tillmann P. Reinhardt T.-C. Paul C. (2000). Networking of near infrared spectroscopy instruments for rapeseed analysis: a comparison of different procedures. J. Near Infrared Spectrosc. 8:101 10.1255/jnirs.269

[B58] Tilman D. Balzer C. Hill J. Befort B. L. (2011). Global food demand and the sustainable intensification of agriculture. Proc. Natl. Acad. Sci. U.S.A. 108, 20260–20264. 10.1073/pnas.1116437108 22106295 PMC3250154

[B59] Tkachuk R. (1981). Oil and protein analysis of whole rapeseed kernels by near infrared reflectance spectroscopy. J. Am. Oil Chem. Soc. 58, 819–822. 10.1007/BF02665588

[B60] Tollenaar M. (1989). Genetic improvement in grain yield of commercial maize hybrids grown in Ontario from 1959 to 1988. Crop Sci. 29, 1365–1371. 10.2135/cropsci1989.0011183X002900060007x

[B61] Wang L. Mühling K.-H. Schulte auf'm Erley S. (2016). Nitrogen efficiency and leaf nitrogen remobilisation of oilseed rape lines and hybrids. Ann. Appl. Biol. 169, 125–133. 10.1111/aab.12286

[B62] Weiser C. Fuß R. Kage H. Flessa H. (2017). Do farmers in Germany exploit the potential yield and nitrogen benefits from preceding oilseed rape in winter wheat cultivation? Arch. Agron. Soil Sci1. 1–13. 10.1080/03650340.2017.1326031

[B63] Wickham H. (2009). ggplot2: Elegant Graphics for Data Analysis. New York, NY: Springer-Verlag.

[B64] Zhao J. Becker H. C. Zhang D. Zhang Y. Ecke W. (2006). Conditional QTL mapping of oil content in rapeseed with respect to protein content and traits related to plant development and grain yield. Theor. Appl. Genet. 113, 33–38. 10.1007/s00122-006-0267-5 16614833

[B65] Zhu G. Peng S. Huang J. Cui K. Nie L. Wang F. (2016). Genetic improvements in rice yield and concomitant increases in radiation- and nitrogen-use efficiency in middle reaches of Yangtze River. Sci. Rep. 6:21049. 10.1038/srep21049 26876641 PMC4753450

