# Supplementary material for: Recent Genetic Gains in Nitrogen Use Efficiency in Oilseed Rape
Source: Front Plant Sci. 2017 Jun 7;8:963. doi: 10.3389/fpls.2017.00963 (PMC5461335; doi:10.3389/fpls.2017.00963)
Supplement: Supplementary file 1 [file Table1.DOCX]

**Supplementary Tables**

**Table S1: Environment specific soil conditions**

| **Environment** | **P_2_O_5_ [mg/100g]** | **K_2_O [mg/100g]** | **Soil pH** | **Soil Type according to FAO,**  **yield index units** | **N_min_ at beginn of vegetation**  **[kg N/ha]** |
| --- | --- | --- | --- | --- | --- |
| ASE15 | 14.8 | 15 | 8.1 | silty loam | 47 |
| ASE16 | 7.5 | 10 | NA | loamy sand, aprox. 45 | 29 |
| BOV16 | 16.0 | 15 | NA | sandy loam, 45 | 21 |
| MOS15 | 10.9 | 13 | 7.1 | loessic loam | 21 |
| MOS16 | 12.0 | 18 | NA | loessic loam | 26 |
| NIE16 | 8.9 | 13 | 6.9 | loam,  68 | 15 |
| RHH15 | 10.4 | 6 | 7.6 | loam,  80 | 25 |
| RHH16 | 10.6 | 7 | 7 | loam,  85 | 13 |
| ROS15 | 14.3 | 13 | 7.0 | very loamy sand,  65 | 21 |
| ROS16 | 14.6 | 18 | 7.8 | very loamy sand,  71 | 14 |

**Table S2: Side specific plot size and row spacing**

| **Location** | **Plot size**  [m^2^] | **Spacing between rows** [cm] |
| --- | --- | --- |
| Asendorf (ASE) | 12.0 | 24 |
| Bovenau (BOV) | 12.0 | 27.4 |
| Moosburg (MOS) | 10.2 | 28 |
| Nienstädt (NIE) | 13.5 | 21 |
| Rauischholzhausen (RHH) | 10.5 | 17.8 |
| Rosenthal (ROS) | 15.7 | 22 |

**Table S3: Descriptive statistics for investigated traits for individual environments**

| **Trait** | **Environment** | **Low nitrogen fertilization** | | | | **High nitrogen fertilization** | | | |  |
| --- | --- | --- | --- | --- | --- | --- | --- | --- | --- | --- |
|  |  | **Min** | **Max** | **Mean** | **CoV** | **Min** | **Max** | **Mean** | **CoV** |  |
| Seed Yield | ASE15 | 3.18 | 5.14 | 4.27 | 0.10 | 2.69 | 5.34 | 4.44 | 0.13 |  |
| [t/ha] | MOS15 | 4.28 | 5.79 | 5.32 | 0.06 | 4.70 | 6.37 | 5.59 | 0.06 |  |
|  | RHH15 | 4.09 | 5.00 | 4.45 | 0.05 | 3.78 | 5.13 | 4.69 | 0.07 |  |
|  | ROS15 | 3.57 | 4.96 | 4.35 | 0.07 | 3.92 | 5.61 | 5.04 | 0.07 |  |
|  | ASE16 | 3.28 | 5.39 | 4.20 | 0.11 | 3.19 | 5.55 | 4.38 | 0.13 |  |
|  | BOV16 | 2.75 | 4.22 | 3.42 | 0.11 | 2.76 | 4.53 | 3.60 | 0.12 |  |
|  | MOS16 | 4.74 | 6.05 | 5.59 | 0.05 | 4.56 | 6.44 | 5.51 | 0.07 |  |
|  | NIE16 | 3.09 | 4.99 | 4.30 | 0.10 | 3.02 | 4.90 | 4.29 | 0.10 |  |
|  | RHH16 | 3.94 | 4.93 | 4.44 | 0.05 | 3.51 | 5.06 | 4.44 | 0.07 |  |
|  | ROS16 | 3.44 | 4.96 | 4.35 | 0.08 | 3.79 | 5.29 | 4.54 | 0.09 |  |
| ProteinConc | ASE15 | 14.27 | 17.56 | 16.10 | 0.06 | 16.16 | 19.09 | 17.55 | 0.05 |  |
| [%] | MOS15 | 14.62 | 17.31 | 15.96 | 0.05 | 17.01 | 19.84 | 18.15 | 0.04 |  |
|  | RHH15 | 11.66 | 13.89 | 12.79 | 0.04 | 13.65 | 16.06 | 14.63 | 0.04 |  |
|  | ROS15 | 13.85 | 16.39 | 15.01 | 0.04 | 16.11 | 18.37 | 17.21 | 0.03 |  |
|  | ASE16 | 14.78 | 17.40 | 15.75 | 0.04 | 16.45 | 19.03 | 17.55 | 0.04 |  |
|  | BOV16 | 14.09 | 16.90 | 15.48 | 0.04 | 16.19 | 18.26 | 17.14 | 0.04 |  |
|  | MOS16 | 15.38 | 18.06 | 16.51 | 0.04 | 16.90 | 19.28 | 18.04 | 0.03 |  |
|  | NIE16 | 14.44 | 16.89 | 15.55 | 0.04 | 15.76 | 17.83 | 16.82 | 0.04 |  |
|  | RHH16 | 13.97 | 16.86 | 15.05 | 0.04 | 16.59 | 19.03 | 17.59 | 0.04 |  |
|  | ROS16 | 15.32 | 17.63 | 16.04 | 0.04 | 16.41 | 18.38 | 17.45 | 0.03 |  |
| OilConc | ASE15 | 41.97 | 47.91 | 44.58 | 0.03 | 40.12 | 46.23 | 43.35 | 0.03 |  |
| [%] | MOS15 | 42.89 | 47.54 | 45.24 | 0.03 | 40.57 | 45.20 | 42.85 | 0.03 |  |
|  | RHH15 | 46.71 | 49.74 | 47.93 | 0.02 | 44.23 | 48.20 | 46.31 | 0.02 |  |
|  | ROS15 | 43.30 | 48.27 | 45.44 | 0.03 | 41.17 | 44.88 | 43.35 | 0.02 |  |
|  | ASE16 | 43.70 | 47.98 | 45.85 | 0.03 | 40.77 | 46.37 | 44.46 | 0.03 |  |
|  | BOV16 | 43.69 | 48.58 | 46.05 | 0.03 | 42.05 | 46.21 | 44.45 | 0.02 |  |
|  | MOS16 | 42.57 | 46.59 | 44.75 | 0.02 | 41.19 | 45.47 | 43.51 | 0.02 |  |
|  | NIE16 | 43.02 | 47.24 | 45.24 | 0.02 | 41.75 | 45.66 | 43.80 | 0.02 |  |
|  | RHH16 | 44.27 | 48.64 | 46.20 | 0.02 | 41.93 | 45.64 | 43.86 | 0.03 |  |
|  | ROS16 | 42.30 | 47.20 | 45.20 | 0.03 | 41.90 | 45.65 | 43.94 | 0.02 |  |
| ProteinY | ASE15 | 0.55 | 0.78 | 0.68 | 0.09 | 0.51 | 0.90 | 0.78 | 0.12 |  |
| [t/ha] | MOS15 | 0.74 | 0.95 | 0.85 | 0.06 | 0.89 | 1.15 | 1.01 | 0.06 |  |
|  | RHH15 | 0.50 | 0.63 | 0.57 | 0.05 | 0.56 | 0.75 | 0.68 | 0.06 |  |
|  | ROS15 | 0.55 | 0.74 | 0.65 | 0.07 | 0.69 | 0.97 | 0.87 | 0.06 |  |
|  | ASE16 | 0.55 | 0.81 | 0.66 | 0.10 | 0.60 | 0.96 | 0.77 | 0.12 |  |
|  | BOV16 | 0.43 | 0.66 | 0.53 | 0.10 | 0.45 | 0.76 | 0.62 | 0.11 |  |
|  | MOS16 | 0.84 | 1.00 | 0.92 | 0.05 | 0.84 | 1.15 | 0.99 | 0.07 |  |
|  | NIE16 | 0.51 | 0.79 | 0.67 | 0.09 | 0.54 | 0.83 | 0.72 | 0.09 |  |
|  | RHH16 | 0.61 | 0.77 | 0.67 | 0.07 | 0.66 | 0.88 | 0.78 | 0.07 |  |
|  | ROS16 | 0.58 | 0.79 | 0.70 | 0.07 | 0.69 | 0.92 | 0.79 | 0.07 |  |
| OilY | ASE15 | 1.34 | 2.38 | 1.91 | 0.12 | 1.08 | 2.36 | 1.93 | 0.15 |  |
| [t/ha] | MOS15 | 1.93 | 2.68 | 2.41 | 0.08 | 2.03 | 2.70 | 2.40 | 0.07 |  |
|  | RHH15 | 1.93 | 2.37 | 2.14 | 0.06 | 1.71 | 2.42 | 2.17 | 0.08 |  |
|  | ROS15 | 1.56 | 2.30 | 1.98 | 0.08 | 1.62 | 2.49 | 2.19 | 0.08 |  |
|  | ASE16 | 1.47 | 2.53 | 1.92 | 0.11 | 1.30 | 2.52 | 1.95 | 0.14 |  |
|  | BOV16 | 1.28 | 1.95 | 1.57 | 0.12 | 1.26 | 2.04 | 1.60 | 0.13 |  |
|  | MOS16 | 2.11 | 2.74 | 2.50 | 0.06 | 2.00 | 2.81 | 2.40 | 0.07 |  |
|  | NIE16 | 1.34 | 2.32 | 1.95 | 0.11 | 1.25 | 2.22 | 1.88 | 0.12 |  |
|  | RHH16 | 1.82 | 2.31 | 2.05 | 0.05 | 1.53 | 2.24 | 1.95 | 0.08 |  |
|  | ROS16 | 1.56 | 2.29 | 1.97 | 0.09 | 1.68 | 2.35 | 2.00 | 0.09 |  |
| ProteinConcDFF | ASE15 | 26.92 | 31.34 | 29.02 | 0.04 | 29.42 | 33.02 | 30.96 | 0.03 |  |
| [%] | MOS15 | 27.29 | 31.75 | 29.13 | 0.03 | 30.59 | 34.78 | 31.76 | 0.03 |  |
|  | RHH15 | 22.95 | 26.96 | 24.58 | 0.03 | 25.90 | 29.96 | 27.24 | 0.03 |  |
|  | ROS15 | 25.55 | 29.67 | 27.49 | 0.03 | 28.67 | 32.03 | 30.39 | 0.03 |  |
|  | ASE16 | 27.99 | 30.87 | 29.05 | 0.03 | 30.37 | 32.96 | 31.60 | 0.02 |  |
|  | BOV16 | 26.79 | 30.45 | 28.70 | 0.03 | 29.26 | 32.16 | 30.84 | 0.02 |  |
|  | MOS16 | 28.74 | 32.18 | 29.90 | 0.03 | 31.01 | 34.13 | 31.94 | 0.02 |  |
|  | NIE16 | 26.70 | 30.21 | 28.41 | 0.03 | 28.68 | 31.61 | 29.99 | 0.03 |  |
|  | RHH16 | 26.61 | 30.32 | 27.92 | 0.03 | 29.65 | 33.39 | 31.37 | 0.03 |  |
|  | ROS16 | 27.87 | 30.99 | 29.26 | 0.03 | 29.49 | 32.91 | 31.12 | 0.03 |  |

Adjusted values of highest (Max) and lowest (Min) performing variety is depicted along with the arithmetic mean of the evaluated variety set. Coefficient of variation (CoV)

**Table S4: Analysis of variance for investigates traits for individual environments**

| **Trait** | **Environment** | **Variety** | **NFL** | **Variety x NFL** |
| --- | --- | --- | --- | --- |
| Seed Yield | ASE15 | *** | ** | * |
| [t/ha] | MOS15 | *** | ** | ** |
|  | RHH15 | * | ** | . |
|  | ROS15 | *** | * |  |
|  | ASE16 | *** | *** | *** |
|  | BOV16 | *** | * |  |
|  | MOS16 | *** |  | *** |
|  | NIE16 | *** |  |  |
|  | RHH16 | *** |  |  |
|  | ROS16 | *** | * |  |
| ProteinConc | ASE15 | *** | ** |  |
| [%] | MOS15 | *** | *** |  |
|  | RHH15 | ** | *** | *** |
|  | ROS15 | *** | ** |  |
|  | ASE16 | *** | *** |  |
|  | BOV16 | *** | ** | ** |
|  | MOS16 | *** | *** |  |
|  | NIE16 | *** | * |  |
|  | RHH16 | *** | ** | . |
|  | ROS16 | *** | ** |  |
| OilConc | ASE15 | *** | * |  |
| [%] | MOS15 | *** | *** |  |
|  | RHH15 | *** | ** | *** |
|  | ROS15 | *** | ** | . |
|  | ASE16 | *** | *** |  |
|  | BOV16 | *** | ** | ** |
|  | MOS16 | *** | ** |  |
|  | NIE16 | *** | * |  |
|  | RHH16 | *** | * | . |
|  | ROS16 | *** | *** |  |
| ProteinY | ASE15 | *** | ** | ** |
| [t/ha] | MOS15 | *** | ** | * |
|  | RHH15 |  | ** |  |
|  | ROS15 | *** | ** |  |
|  | ASE16 | *** | *** | *** |
|  | BOV16 | *** | ** | . |
|  | MOS16 | *** | *** | *** |
|  | NIE16 | *** | . |  |
|  | RHH16 | *** | . | . |
|  | ROS16 | *** | *** |  |
| OilY | ASE15 | *** |  | . |
| [t/ha] | MOS15 | *** |  | *** |
|  | RHH15 | ** |  | * |
|  | ROS15 | *** | . |  |
|  | ASE16 | *** |  | *** |
|  | BOV16 | *** |  |  |
|  | MOS16 | *** | * | ** |
|  | NIE16 | *** |  |  |
|  | RHH16 | *** | . |  |
|  | ROS16 | *** |  |  |
| ProteinConc | ASE15 | *** | ** |  |
| in oilfree fraction | MOS15 | *** | *** |  |
| [%] | RHH15 | *** | *** | *** |
|  | ROS15 | *** | *** |  |
|  | ASE16 | *** | *** |  |
|  | BOV16 | *** | ** | * |
|  | MOS16 | *** | *** |  |
|  | NIE16 | *** | * |  |
|  | RHH16 | *** | ** | . |
|  | ROS16 | *** | ** |  |

NFL: Nitrogen fertilization level. Level of significance is indicated by . for p<0.1, * for p<0.01, ** for p<0.005 and *** for p<0.001.
