# Supplementary material for: Recent Genetic Gains in Nitrogen Use Efficiency in Oilseed Rape
Source: Front Plant Sci. 2017 Jun 7;8:963. doi: 10.3389/fpls.2017.00963 (PMC5461335; doi:10.3389/fpls.2017.00963)
Supplement: Supplementary file 2 [file Image1.PDF]

Stahl et al., Supplementary Figures:

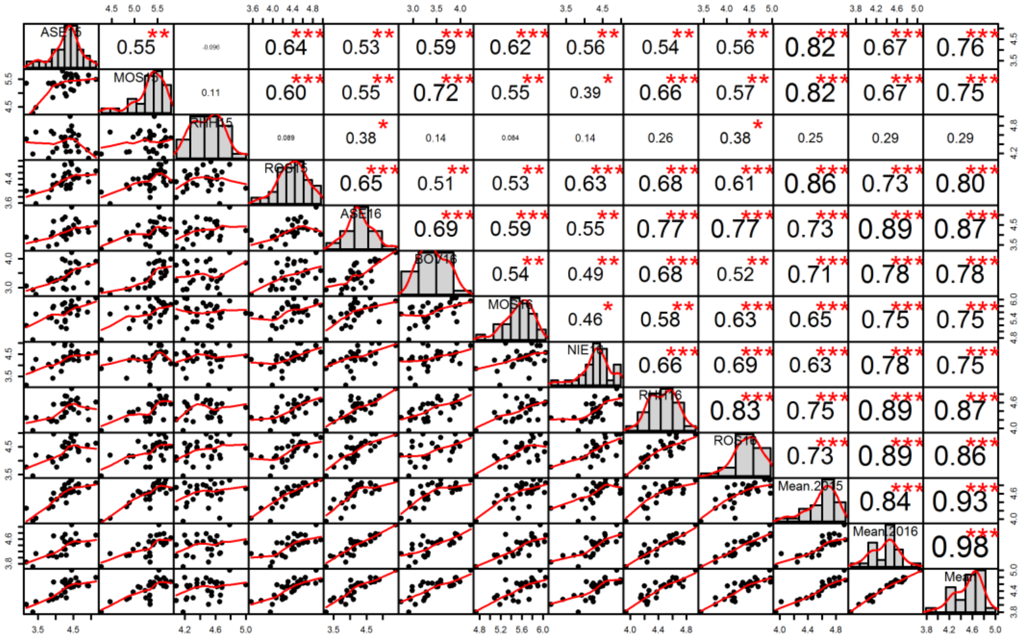

A

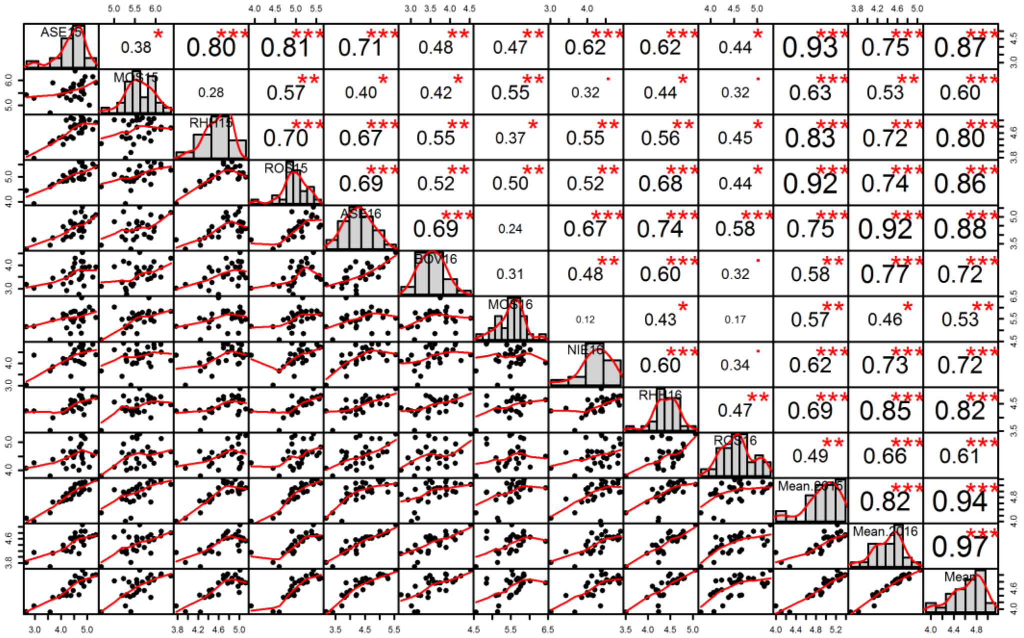

B

Figure S1: Correlation between individual environments for seed yield at low nitrogen fertilization (A) and high nitrogen fertilization (B). Three columns on the right and three rows at the bottom indicate correlations with adjusted means of the year 2015, 2016 and adjusted means across all 10 environments.

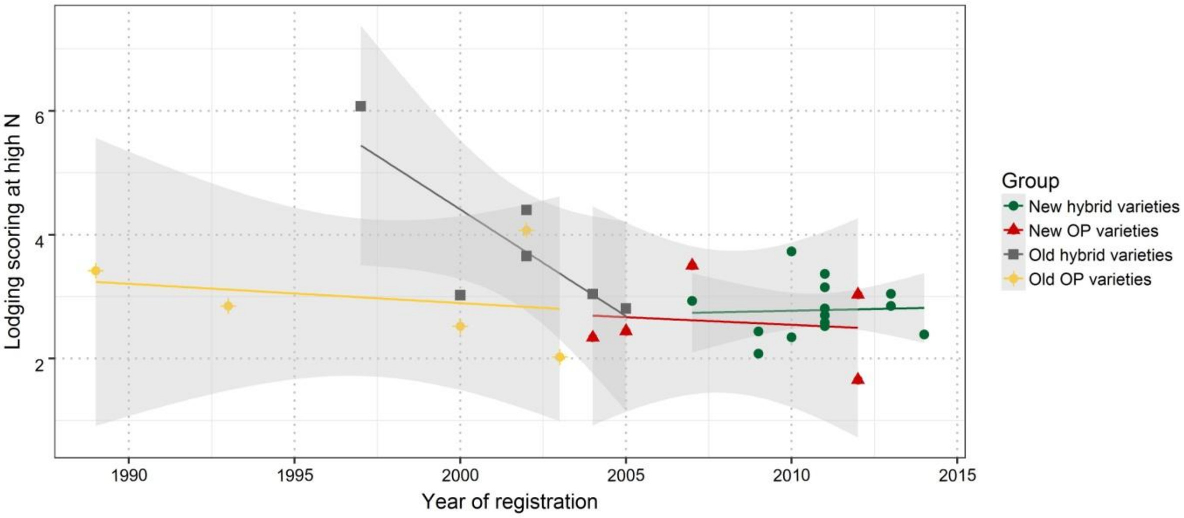

Figure S2: Correlation between year of registration and lodging scoring at high nitrogen fertilization (1-9). Data refer to environment ASE15, ASE16, NIE16 and MOS16.

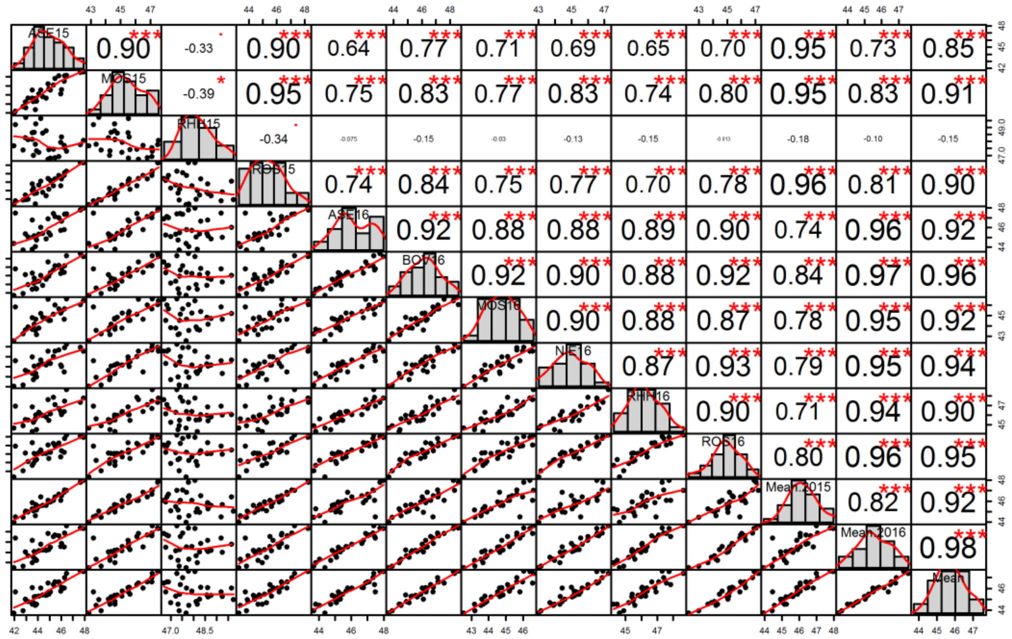

A

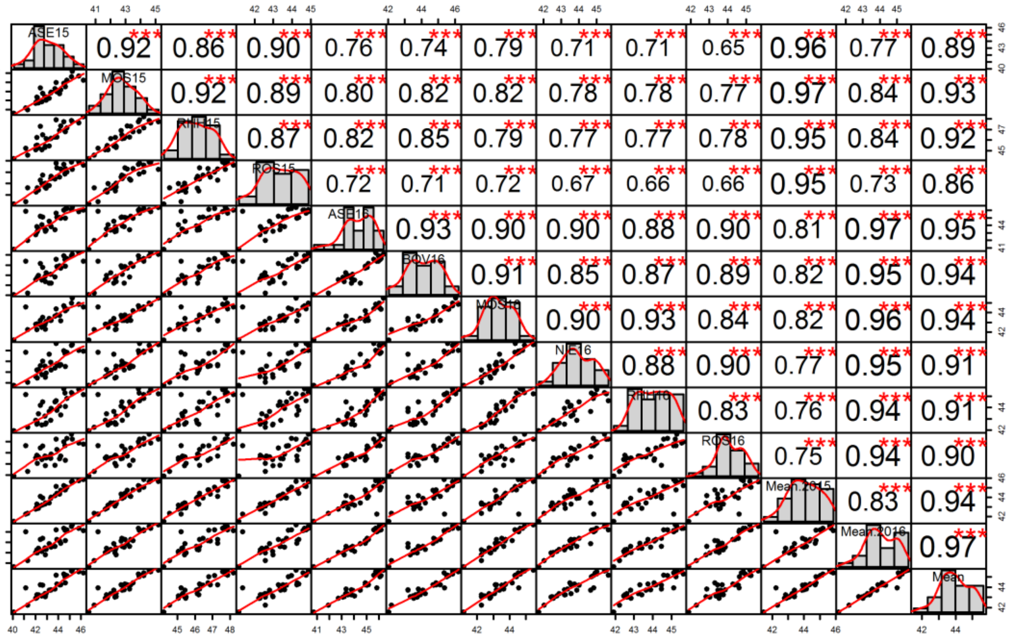

B

Figure S3: Correlation between individual environments for oil concentration at low nitrogen fertilization (A) and high nitrogen fertilization (B). Three columns on the right and three rows at the bottom indicate correlations with adjusted means of the year 2015, 2016 and adjusted means across all 10 environments.

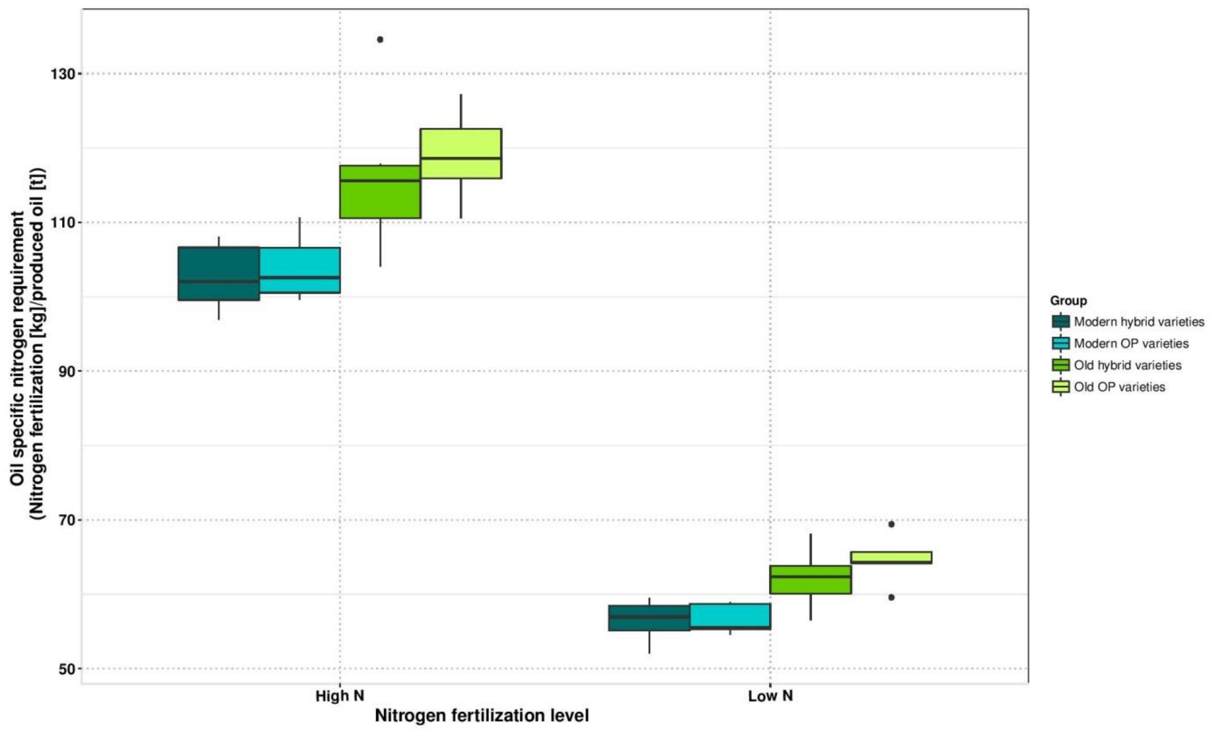

Figure S4: Boxplots for oil specific nitrogen requirement under high (left) and low (right) nitrogen fertilization for individual variety groups.

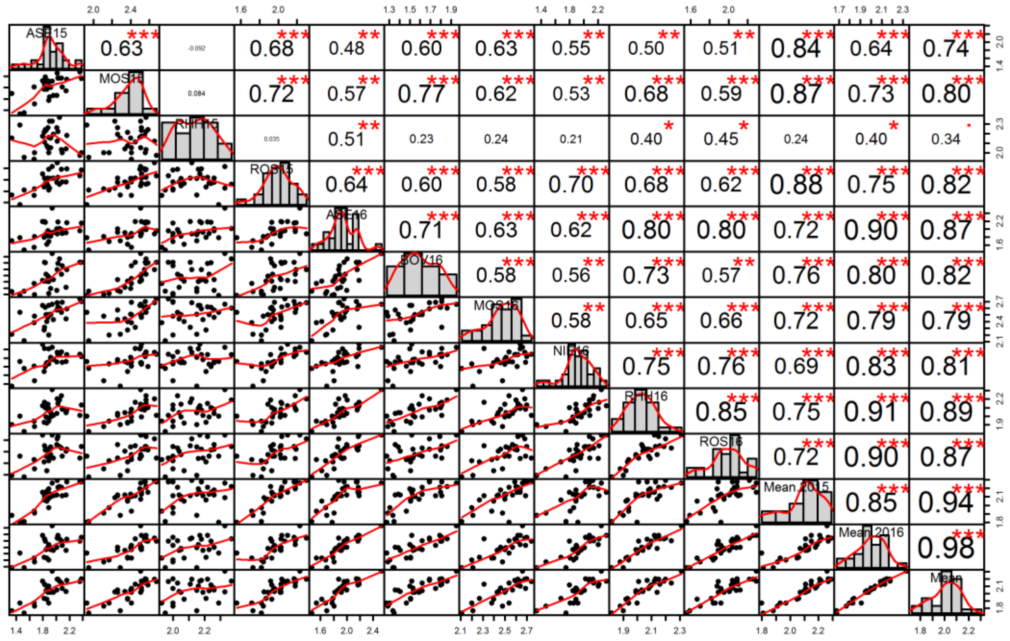

A

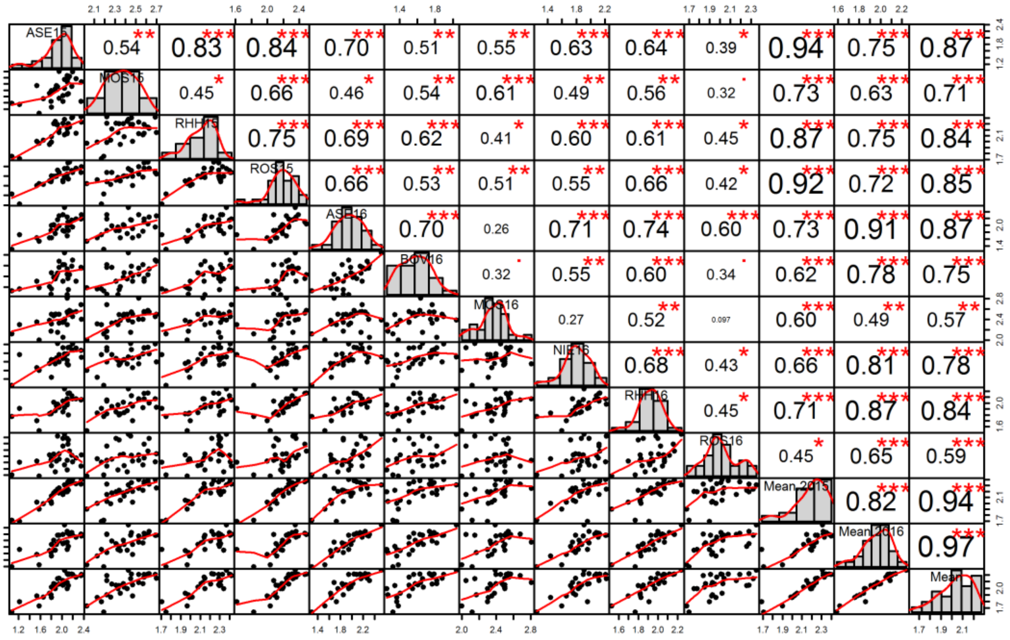

B

Figure S5: Correlation between individual environments for oil yield at low nitrogen fertilization (A) and high nitrogen fertilization (B). Three columns on the right and three rows at the bottom indicate correlations with adjusted means of the year 2015, 2016 and adjusted means across all 10 environments.

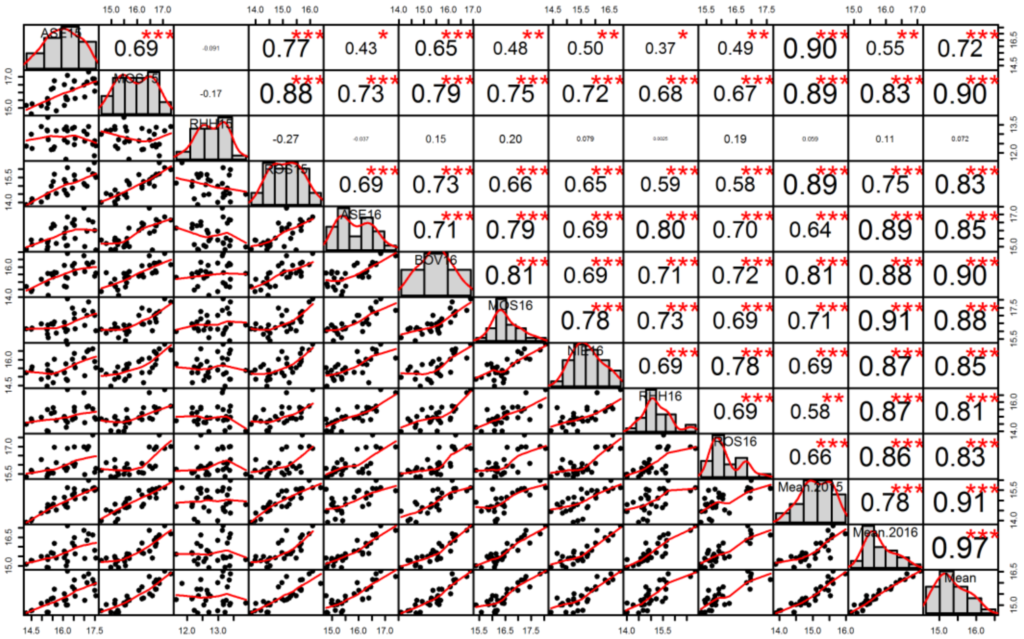

A

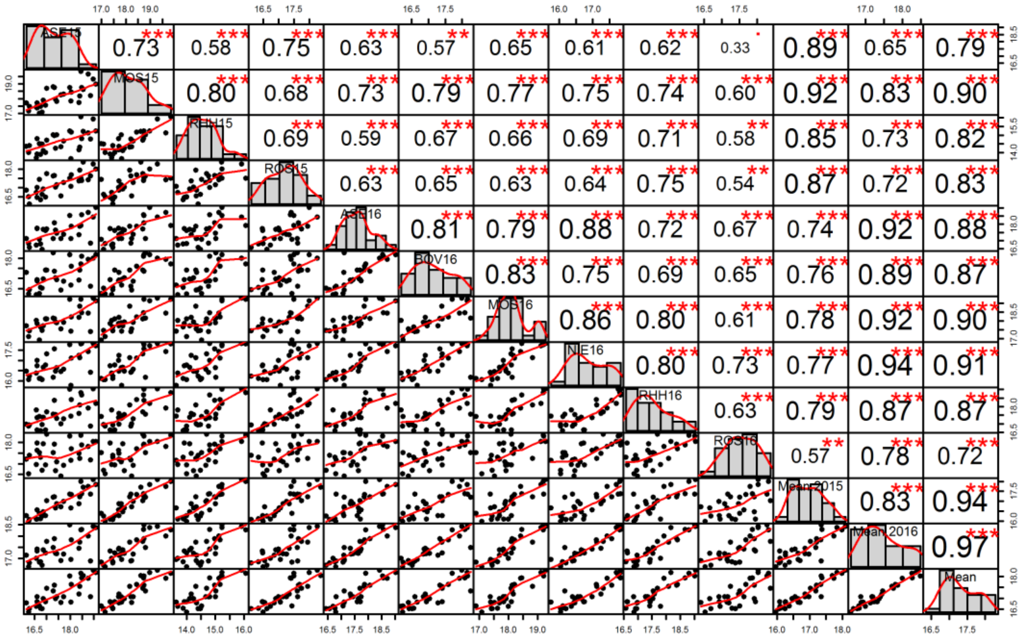

B

Figure S6: Correlation between individual environments for protein concentration at low nitrogen fertilization (A) and high nitrogen fertilization (B). Three columns on the right and three rows at the bottom indicate correlations with adjusted means of the year 2015, 2016 and adjusted means across all 10 environments.

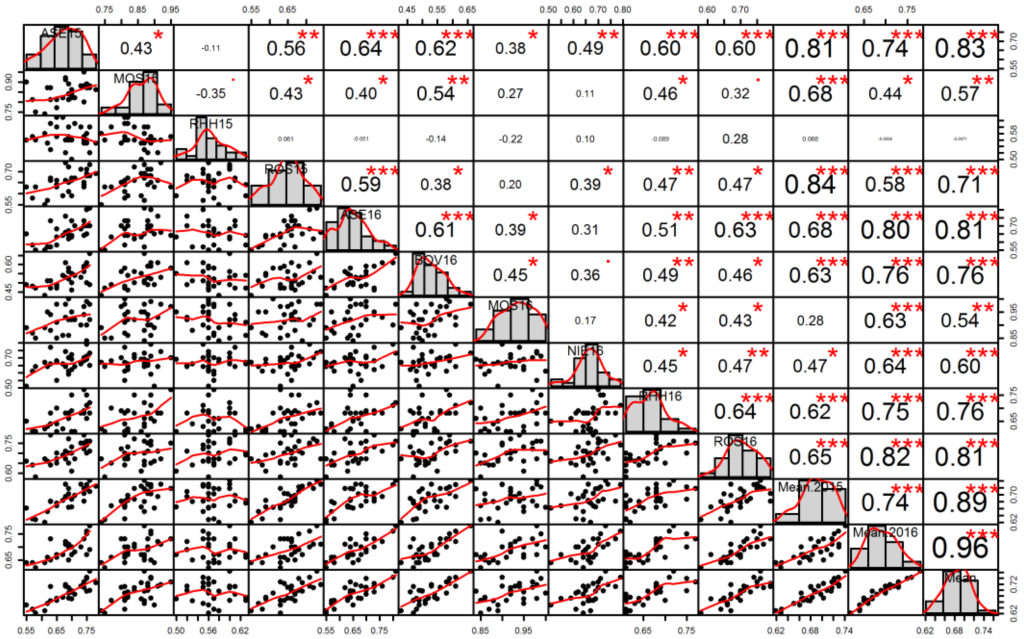

A

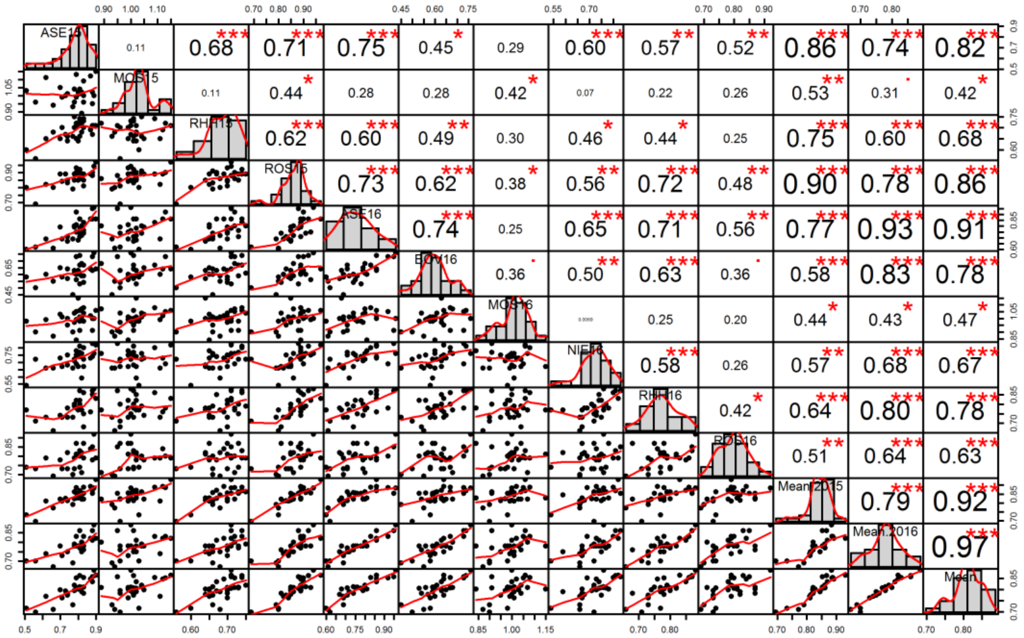

B

Figure S7: Correlation between individual environments for protein yield at low nitrogen fertilization (A) and high nitrogen fertilization (B). Three columns on the right and three rows at the bottom indicate correlations with adjusted means of the year 2015, 2016 and adjusted means across all 10 environments.
